# North European invasion by common ragweed is associated with early flowering and dominant changes in *FT/TFL1* expression

**DOI:** 10.1093/jxb/ery100

**Published:** 2018-03-14

**Authors:** Lejon E M Kralemann, Romain Scalone, Lars Andersson, Lars Hennig

**Affiliations:** 1 Department of Plant Biology and Linnean Center for Plant Biology, Swedish University of Agricultural Sciences, Uppsala, Sweden; 2 Department of Crop Production Ecology, Uppsala Ecology Center, Swedish University of Agricultural Sciences, Uppsala, Sweden

**Keywords:** *Ambrosia artemisiifolia*, common ragweed, *FLOWERING LOCUS T*, flowering time, invasive species, phosphatidylethanolamine-binding protein, *TERMINAL FLOWER 1*

## Abstract

During the last two centuries, the North American common ragweed (*Ambrosia artemisiifolia* L.) invaded a large part of the globe. Local adaptation of this species was revealed by a common garden experiment, demonstrating that the distribution of the species in Europe could extend considerably to the North. Our study compares two populations of common ragweed (one from the native range and one from the invaded range) that differ in flowering time in the wild: the invasive population flowers earlier than the native population under non-inductive long-day photoperiods. Experiments conducted in controlled environments established that the two populations differ in their flowering time even under inductive short-day photoperiods, suggesting a change in autonomous flowering control. Genetic analysis revealed that early flowering is dominantly inherited and accompanied by the increased expression of the floral activator *AaFTL1* and decreased expression of the floral repressor *AaFTL2*. Early flowering is also accompanied by reduced reproductive output, which is evolutionarily disadvantageous under long vegetation periods. In contrast, under short vegetation periods, only early-flowering plants can produce any viable seeds, making the higher seed set of late-flowering plants irrelevant. Thus, earlier flowering appears to be a specific adaptation to the higher latitudes of northern Europe.

## Teaser

North European invasion by common ragweed is associated with earlier flowering, dominant changes in *FT/TFL1* expression, and reduced seed production.

## Introduction

Common ragweed (*Ambrosia artemisiifolia* L.) is an annual invasive plant with a negative impact on the economy due to its role as an agricultural weed (Weaver, 2001; Essl *et al.*, 2015) and as a source of allergenic wind-dispersed pollen grains (Arbes *et al.*, 2005). As a typical short-day (SD) plant, *A. artemisiifolia* starts to produce flowers when daily photoperiods drop below a critical threshold (~14 h of light) (Deen *et al.*, 1998; Essl *et al.*, 2015). The first sign of flowering is a pale green bud at the shoot apex, forming the main inflorescence (see Supplementary Fig. S1 at *JXB* online for a scheme of reproductive development of *A. artemisiifolia* and images of the plant). This inflorescence grows to become a leafless raceme with dozens of staminate (male) flower heads (Essl *et al.*, 2015). At the same time, pistillate (female) flowers develop at the axils of the upper leaves and at the involucres of the male inflorescences (Essl *et al.*, 2015). Maturity of male flowers is indicated by the thick yellow pollen-coated anthers protruding from the male flowers, while female maturity is indicated by the protrusion of a dichotomous stigma out of each female flower (Essl *et al.*, 2015). Fertilized female flowers develop a fruit that mostly consists of a single seed (Essl *et al.*, 2015).

The well-documented invasion history of *A. artemisiifolia* makes this species a good model to study eudicot invasiveness. *Ambrosia artemisiifolia* is native to North America and has successfully spread to all continents except Antarctica during the last two centuries (Gaudeul *et al.*, 2011). The first European record is from France in 1863 (Chauvel *et al.*, 2006), and the first Australian record is from 1911 (Webb, 1987). Although *A. artemisiifolia* seeds can be naturally dispersed by epizoochory (Rosas *et al.*, 2008), most of its dispersal in the last centuries is probably due to human activity: *A. artemisiifolia* seeds are routinely detected in imported bird feed and livestock fodder (EFSA, 2010), and its settlement follows anthropic land disturbance (Essl *et al.*, 2009). In Europe, the main infestations can be found in South-East Europe (particularly Hungary, Croatia, and Serbia), although *A. artemisiifolia* plants were observed from Great Britain to western Russia and from southern Italy to southern Sweden (Buters *et al.*, 2015; Essl *et al.*, 2015). Stable populations, however, can form in northern Europe only if mechanisms evolve to cope with the early onset of low temperatures in autumn. SD plants such as *A. artemisiifolia* cannot easily spread to higher latitudes. At higher latitudes, temperatures are too low for growth and for seed development soon after day length drops under the critical threshold to induce flowering. Therefore, cold resistance or accelerated flowering is required for successful seed production under these conditions. Changing flowering time is particularly likely to underlie rapid adaptation, because it can cause reproductive isolation. The gene pool of a population in the northern part of the invaded range would be less affected by non-adaptive alleles from the mother population adapted to lower latitudes. Previous common garden experiments have identified a latitudinal cline in flowering time among invasive European *A. artemisiifolia* populations, indicating that indeed a change in flowering time has occurred to adapt to higher latitudes (Leiblein-Wild and Tackenberg, 2014; Scalone *et al.*, 2016).

In flowering plants, flowering time is regulated by members of the phosphatidylethanolamine-binding protein (*PEBP*) gene family (Bradley *et al.*, 1996; Kardailsky *et al.*, 1999; Yano *et al.*, 2001; Lifschitz *et al.*, 2006; Blackman *et al.*, 2010). *PEBP* genes are found in all kingdoms of life, and in plants three major clades exist: *FLOWERING LOCUS T*-like (*FT*) genes, *TERMINAL FLOWER 1*-like (*TFL1*) genes, and *MOTHER OF FT AND TFL1*-like (*MFT*) genes. The *MFT* clade is the oldest of the three, and *MFT*-like genes are found in lycophytes, bryophytes, gymnosperms, and angiosperms, where they have a function in seed germination (Karlgren *et al.*, 2011). *FT/TFL1* genes are absent in studied lycophytes and bryophytes, but were detected in all studied gymnosperms and angiosperms (Karlgren *et al.*, 2011). Separate *FT* and *TFL1* clades originate in gymnosperms and further diverged in angiosperms (Liu *et al.*, 2016). Both clades contain genes that regulate flowering time (Bradley *et al.*, 1996; Kardailsky *et al.*, 1999), although genes of that family can also have other functions such as regulating bulb or tuber formation (Navarro *et al.*, 2011; Lee *et al.*, 2013), stomatal opening (Ando *et al.*, 2013), inflorescence architecture (Bradley *et al.*, 1996), and flower morphology (Molinero-Rosales *et al.*, 2004).

While most *FT/TFL1* genes function as floral activators or repressors, some have no effect on flowering time (e.g. Lee *et al.*, 2013). FT/TFL1 proteins often act in a non-cell-autonomous manner (Lifschitz *et al.*, 2006; Corbesier *et al.*, 2007; Mathieu *et al.*, 2007; Tamaki *et al.*, 2007; Navarro *et al.*, 2011). In particular, Arabidopsis FT and its rice homologue Hd3a are produced in leaves and move through the phloem to the shoot apex (Corbesier *et al.*, 2007; Jaeger and Wigge, 2007; Mathieu *et al.*, 2007) where they interact with the bZIP transcription factor FLOWERING LOCUS D (FD) and together regulate, among others, the floral integrator gene *SOC1* and the floral meristem identity gene *FUL* to induce flowering (Abe *et al.*, 2005; Wigge *et al.*, 2005; Yoo *et al.*, 2005). Some FT-like proteins act as floral repressors, and the balance of activators and repressors determines flowering time (Higuchi *et al.*, 2013), presumably through competition for FD (Ahn *et al.*, 2006; Ryu *et al.*, 2014). Many *FT*/*TFL1* genes are floral integrators at which multiple flowering pathways converge. In Arabidopsis, rice, barley, and potato, for instance, *FT/TFL1* genes are activated by photoperiod via CONSTANS-like (COL) proteins (An *et al.*, 2004; Kikuchi *et al.*, 2011; Abelenda *et al.*, 2016; Nemoto *et al.*, 2016). Similar to *FT/TFL1*, *COL* genes are of ancient origin, occurring in all land plants and some algal lineages (Valverde, 2011). It is likely that most plants possess COL–FT/TFL1 modules, but the specifics of the regulation of *FT/TFL1* genes differ between species. *COL* transcript and protein levels are regulated by light in long-day (LD) Arabidopsis, but not in SD rice (Nemoto *et al.*, 2016; Shim *et al.*, 2017). Arabidopsis CO activates *FT/TFL1* genes, while the rice homologue of CO can activate or repress *FT/TFL1* genes depending on its photoperiod-regulated interaction partners (Nemoto *et al.*, 2016). In addition to photoperiod, Arabidopsis *FT* is also a main target of the vernalization pathway, which responds to long periods of cold such as during winter (Ream *et al.*, 2012). Similarly, in wheat and barley, *FT* genes integrate photoperiod and vernalization signals promoting flowering (Ream *et al.*, 2012). In strawberry, *FT/TFL1* genes integrate light quality, temperature, and photoperiod (Rantanen *et al.*, 2014; Nakano *et al.*, 2015). In trees and perennial herbs, *FT/TFL1* genes may also convey competence to flower in relation to plant age (Böhlenius *et al.*, 2006; Hsu *et al.*, 2006; Wang *et al.*, 2011). Together, *FT/TFL1* genes serve as central developmental regulators in many plant species and constitute a major potential target of evolution in the adaptation to particular environmental conditions.

In this study, we asked whether the previously identified *A. artemisiifolia* populations that flowered differently in a common garden show a similar difference in flowering time in a controlled environment differing only in photoperiod. We studied the genetic basis of the flowering time of *A. artemisiifolia* by creating an F_1_ hybrid population and by studying candidate genes involved in flowering time regulation. We also studied the reproductive output to estimate the evolutionary significance and potential fitness benefits or costs of the early flowering trait.

## Materials and methods

### Plant material

A previous common garden study (Scalone *et al.*, 2016) identified two *A. artemisiifolia* L. populations with significantly different flowering times: one early-flowering population from the northern part of the invaded European range (Drebkau, Germany N51°38'21'' E14°11'50''), the ‘invasive population’, and one late-flowering population from a central part of the native range (Lexington, KY, USA N38°01' W84°33'), the ‘native population’.

Seeds from at least 10 different plants in the two field populations were used to generate the respective study populations. For an additional analysis [gene expression at 15 days after germination (DAG)], a new seed batch was generated representing the original genetic diversity of the populations sampled from the wild by performing intrapopulation crosses (native×native and invasive×invasive) of all members of one population with each other. The intrapopulation F_1_ seeds were used in the experiment. For tests of the genetic basis for different flowering time, interpopulation F_1_ seeds (native×invasive) were used.

For the expression of the *A. artemisiifolia* FT/TFL1-like (FTL) proteins in *Arabidopsis thaliana* (L.) Heynh., coding sequences were amplified from cDNA and cloned into pEARLEYGATE100 (Earley *et al.*, 2006). Transformation of Col-0 wild-type plants was performed by floral dip, and seeds were germinated on medium containing phosphinothricin. Plants transformed with empty pMDC160 (Brand *et al.*, 2006) served as the phosphinothricin-resistant control. Seedlings were transferred to soil at 6 DAG. For expression analyses in Arabidopsis, phosphinothricin-grown T_2_ plants were used.

### Growth conditions

*Ambrosia artemisiifolia* seeds were stratified on moist filter paper for 4 weeks and subsequently placed in a growth chamber providing 16 h light of 50 µmol m^–2^ s^–1^ at 27 °C and 8 h darkness at 15 °C to induce germination. After germination, seedlings were transplanted to pots with pumice (0.5 mm<Ø>2.8 mm; Hekla Green, Bara Mineraler, Bara, Sweden), and moved into a climate chamber with SD conditions (12 h light of 280 µmol m^–2^ s^–1^ at 25 °C; 12 h darkness at 15 °C; humidity at 70%). Plants were watered with nutrient-enriched water (2 ml l^–1^ of Wallco växtnäring 53-10-43+micro from Cederroth, Upplands Väsby, Sweden). The position of the plants in the chamber was randomized daily. Arabidopsis plants were grown under LD conditions (16 h light of 220 µmol m^–2^ s^–1^ at 22 °C; 8 h darkness at 20 °C).

### Measuring flowering time

Once a day, *A. artemisiifolia* plants were inspected for signs indicating the start of specific phenological phases (see Supplementary Fig. S1 for a scheme of reproductive development of *A. artemisiifolia*): (i) appearance of the main male inflorescence bud (start of male floral initiation); (ii) first release of yellow pollen grains (end of male maturation phase); and (iii) first full extension of a dichotomous stigma out of a female flower (end of female maturation phase). The phases are referred to as ‘vegetative-male initiation’ (from germination to male floral initiation; symbol: ♂1), ‘male maturation’ (from male floral initiation to first pollen release; symbol: ♂2) and ‘vegetative-female maturation’ (from germination to appearance of the first elongated pistil; symbol: ♀). Plants with only pistillate flowers were excluded from the analysis. Because data were not normally distributed, the Mann–Whitney U-test (Marx *et al.*, 2016) was used to test for significant differences between means.

Arabidopsis plants were inspected daily for the appearance of the first inflorescence (time to bolting, which was here equated with floral initiation). Upon bolting, both the date and number of (true) rosette leaves were noted.

### Other plant measurements

Final plant size characteristics were determined: plant height (length of the main stem, not including the male inflorescence), basal diameter of the main stem, number of branches on the main stem, root weight, and root area (measured using a camera operated by WD3 WinDIAS software, Delta-T Devices, Cambridge, UK). For the last 2 months of the experiment, seeds were collected weekly. For each plant, the number of produced fruits was counted and the total fruit weight was measured. Mann–Whitney U-tests were performed to test for significant differences between groups.

### Identifying *A. artemisiifolia FT/TFL1* genes

Arabidopsis and *Helianthus annuus FT/TFL1* cDNA sequences were used as queries in BLAST searches of expressed sequence tag (EST) libraries (Lai *et al.*, 2012) from two *A. artemisiifolia* populations (one from the USA and one from Hungary) and four *A. trifida* populations (two from the USA and two from China). The two *FT/TFL1-l* gene fragments thus obtained were used for rapid amplification of cDNA ends (RACE) to acquire the full-length sequences. Another partial *FT/TFL1* gene fragment reported by Li *et al.* (2015) was amplified using the published primers and also used for RACE. The three identified *A. artemisiifolia FT/TFL1* genes were designated *AaFTL1*, *AaFTL2*, and *AaFTL3*.

### RACE

5'- and 3'-RACE were performed using the GeneRacer kit (Invitrogen, Carlsbad, CA, USA), according to the manufacturer’s instructions using cDNA based on RNA from plants of the native population. The optional nested PCRs were always included. RACE products were cloned into pJET1.2 and sequenced. The 5' end of the Aa*FTL3* transcript could not be obtained, presumably due to low transcript abundance. *Ambrosia artemisiifolia FT/TFL1* sequences were compiled from multiple sequencing runs, each base confirmed by at least three independent sequencing reactions. Supplementary Table S1 lists the RACE primers. Supplementary Table S2 lists the obtained *A. artemisiifolia FT/TFL1* cDNA sequences with GenBank accession numbers.

### Phylogenetic analysis

For phylogenetic analysis, amino acid sequences were obtained by searching the literature for FT/TFL1 proteins with confirmed function. FT/TFL1 proteins from the same species but with unknown function were included. This resulted in a list of FT/TFL1 proteins from 33 species and 16 families. FT/TFL1 proteins were designated as floral activators/repressors if overexpression resulted in earlier/later flowering or if knock-out resulted in late/early flowering, respectively. Sequences were considered as flowering neutral if their overexpression or mutation did not result in a change in flowering time. Sequences of most FT/TFL1 proteins were from NCBI; the remaining sequences were from other databases or studies (Supplementary Tables S3, S4). Sequences were aligned using MUSCLE with the maximum number of iterations set to 16 (Edgar, 2004; Dereeper *et al.*, 2008), and curated using GBLOCKS allowing gaps (Dereeper *et al.*, 2008). Curated sequences were used in a Maximum Likelihood analysis (PhyML) (Dereeper *et al.*, 2008; Guindon *et al.*, 2010), allowing gaps and using the WAG substitution model. Branch confidence was determined by aLRT (Guindon *et al.*, 2010), and branches with a confidence score of <85% were collapsed. The analysis involved 172 amino acid sequences with a total of 132 sequence positions. For additional confirmation, a minimum evolution analysis as implemented in MEGA 7.0.20 (Kumar *et al.*, 2016) was performed. A consensus tree was made of 100 bootstrap replicates, and the branches occurring in <50% of replicates were collapsed.

### Prediction of FT/TFL1 protein function

Curated aligned sequences were used to generate consensus sequences to identify amino acid residues unique to functional groups (floral activators, repressors, or flowering neutral proteins) within either the FT clade or the TFL1 clade. The *A. artemisiifolia* sequences were compared with these consensus sequences. As an additional separate analysis, a score was determined to link protein functionality with the amino acid residue diversity across the entire curated aligned sequences. This involved creation of first a score for each position based on the prevalence of different amino acid residues within the group of activators/repressors and non-activators/non-repressors (for FT-like proteins/TFL1-like proteins, respectively). This is a value between 1 (occurs in all of the functional group) and 0 (occurs in none of the functional group). Then each FT/TFL1 sequence was investigated separately and the aforementioned values were assigned to each position. For each sequence, the values of individual positions were averaged to form a score for the entire sequence. Each sequence then results in an activator/repressor score and a non-activator/non-repressor score (again for FT-like proteins/TFL1-like proteins, respectively), and the ratio between these scores is the value presented (named ‘activator ratio’ and ‘repressor ratio’). Cut-off values were determined empirically to minimize the probability of falsely calling a known non-activator an activator, or a known non-repressor a repressor.

### Gene expression

*Ambrosia artemisiifolia* leaf tip and shoot apex samples were collected at 4 h Zeitgeber time (ZT). Arabidopsis seedlings were collected at 8 h ZT. RNA extraction was performed using an RNeasy plant mini kit (Qiagen, Hilden, Germany) according to the manufacturer's instructions. Genomic DNA was removed with the RNase-free DNase set (Qiagen) following the optional on-column DNase digestion protocol. cDNA synthesis was performed with the RevertAid First Strand cDNA synthesis kit (Thermo Scientific). HOT FIREPol polymerase and Evagreen qPCR Mix Plus (Solis Biodyne, Tartu, Estonia) was used in conjunction with the MyIQ Single-color real-time PCR detection system (Biorad, Hercules, CA, USA) for transcript quantification. For Arabidopsis quantitative reverse transcription–PCRs (RT–qPCRs), *PP2AA3* (*AT1G13320*) was used as the reference gene (Mozgová *et al.*, 2015). For the *A. artemisiifolia* RT–qPCRs, the following reference genes were used: *TUBULIN A* (*TUA*) (El Kelish *et al.*, 2014), *TUBULIN B* (*TUB*) (Li *et al.*, 2015), and *GAPDH* (Hodgins *et al.*, 2013). Because *TUB* was found to be the most stable across samples (Supplementary Table S5), expression values relative to *TUB* are shown in the main figures. Expression values relative to the other reference genes are shown in the Supplementary figures. The observed trends were consistent and independent of the choice of reference gene. Ct values of technical replicates (three per sample) were used to calculate the mean normalized expression (MNE) according to Simon (2003). Then the MNEs of all biological replicates were used to calculate the ‘average relative expression’. For most *A. artemisiifolia* gene expression values, biological replicates were single individuals from the same population, but for the 15 DAG time point and in the leaf versus shoot apex expression analysis, four pools of four individuals each were used. For Arabidopsis transformants, each biological replicate was an independent transgenic line pooling 10 individuals per line. For experiments where all data met normal and equal variance criteria, a one-tailed *t*-test was performed; otherwise a Mann–Whitney U-test was performed.

## Results

### An invasive population of *A. artemisiifolia* from Germany has a shorter vegetative phase

**Fig. 1.**
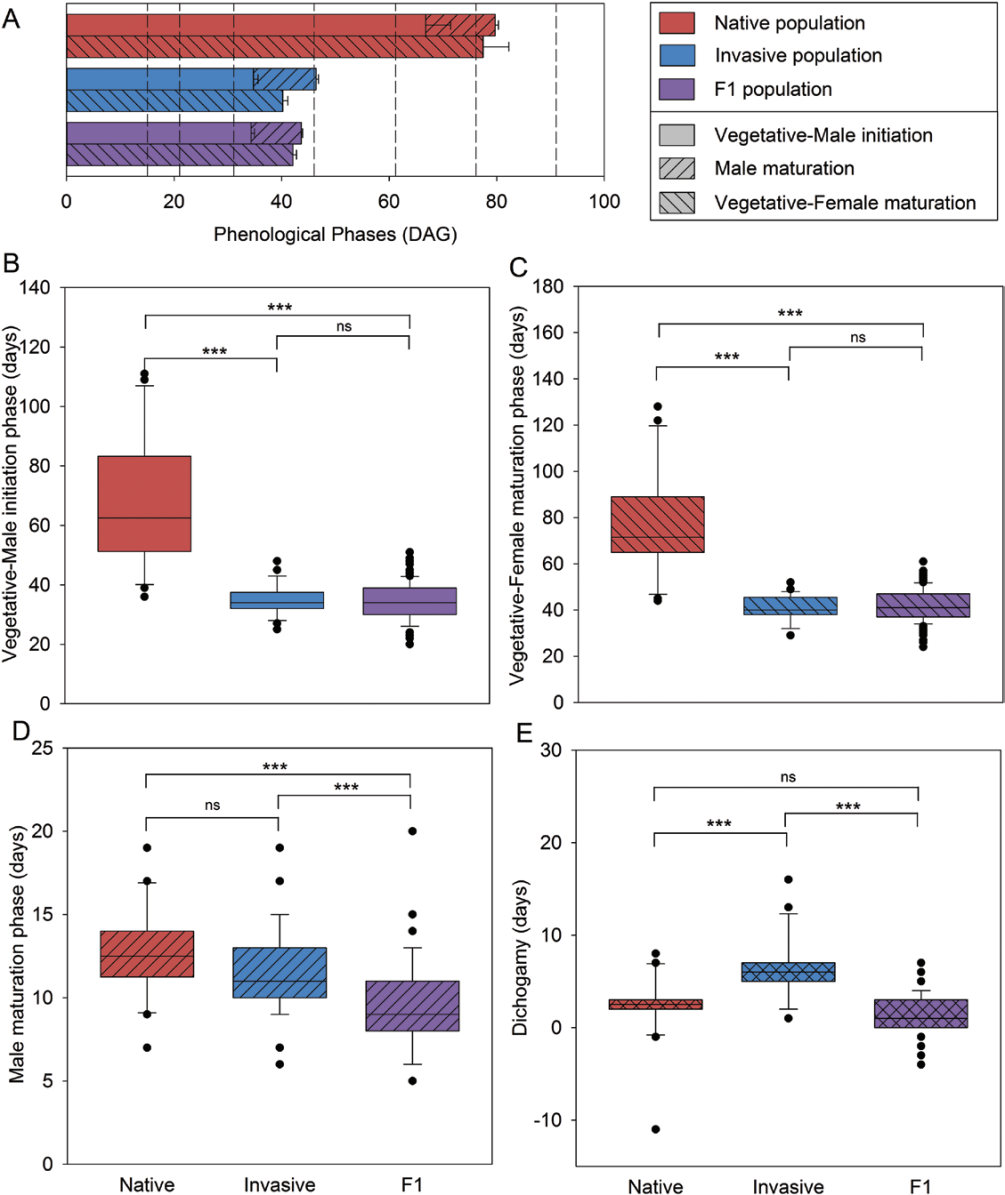
Flowering phenology of the native, invasive, and F_1_ populations. (A) Averages of the different phenological phases together for the native population (red, *n*=20), the invasive population (blue, *n*=36), and the F_1_ population (purple, *n*=111). Dashed vertical lines indicate times of sample collection for gene expression analysis. (B) Length of the vegetative-male initiation phase. (C) Length of the vegetative-female maturation phase. (D) Length of the male maturation phase. (E) Degree of dichogamy, expressed by the time between the appearance of the first mature male flower and the appearance of the first mature female flower. Box plots show the first, second, and third quartiles (the box), the 10th and 90th percentiles (the whiskers), and outliers as individual dots. Mann–Whitney U-tests were performed to test for significant differences between groups (ns *P*>0.05, ***P<0.001; Bonferroni correction was applied with *m*=12 to the α of individual comparisons to obtain the indicated overall α values).

A previous study (Scalone *et al.*, 2016) reported that an invasive *A. artemisiifolia* population flowered earlier than a native population, when both were grown in the same experimental garden with naturally changing photoperiod (see Supplementary Fig. S1 for images of plants from the two populations). The present study extended this experiment using controlled cultivation conditions and constant SD photoperiods. For the tested native and invasive populations, the lengths of three phenological phases were recorded (Fig. 1A). The plants from the invasive population had shorter ‘vegetative-male initiation’ and ‘vegetative-female maturation’ phases ($\bar{\text{X}}$_n♂1_=66.8 d, $\bar{\text{X}}$_i♂1_=34.8 d, *P*<0.0001, Fig. 1B; $\bar{\text{X}}$_n♀_=77.5 d, $\bar{\text{X}}$_i♀_=40.2 d, *P*<0.0001, Fig. 1C), but the ‘male maturation’ phase was not significantly different ($\bar{\text{X}}$_n♂2_=12.9 d, $\bar{\text{X}}$_i♂2_=11.7 d, *P*=0.064, Fig. 1D). Invasive plants also showed an increased degree of dichogamy, namely delayed development of female after male flowers ($\bar{\text{X}}$_n♂♀_=2.2 d, $\bar{\text{X}}$_i♂♀_=6.2 d, *P*<0.0001, Fig. 1E).

In addition to these two populations, a third population was investigated: the F_1_ of native×invasive crosses. For these plants, all phases were shorter than for the parental native plants ($\bar{\text{X}}$_F1♂1_=34.3 d, $\bar{\text{X}}$_F1♂2_=9.3 d, $\bar{\text{X}}$_F1♀_=42.1 d, *P*<0.0001, Fig. 1B–D). The F_1_ plants had ‘vegetative-male initiation’ and ‘vegetative-female maturation’ phases equally short as those of plants from the invasive population (*P*=0.59 and *P*=0.16, respectively, Fig. 1B, C), indicating that the invasive population has contributed (a) dominant allele(s) controlling these traits. The ‘male maturation’ phase of the F_1_ plants was shorter not only than in the native population, but also than in the invasive population (*P*<0.0001; Fig. 1D). The reasons for this pattern could be overdominance of an ‘early’ allele for this trait or genetic heterogeneity in a parent population that is lost in the experimental F_1_ plants. Unlike the invasive plants, the F_1_ plants did not show increased dichogamy ($\bar{\text{X}}$_F1♂♀_=1.6 d, *P*=0.02, Fig. 1E). In short, there appears to be at least one (over-) dominant allele that makes the invasive plants flower earlier than the native plants by shortening the vegetative phase.

### Early flowering is associated with a cost in reproductive output

**Fig. 2.**
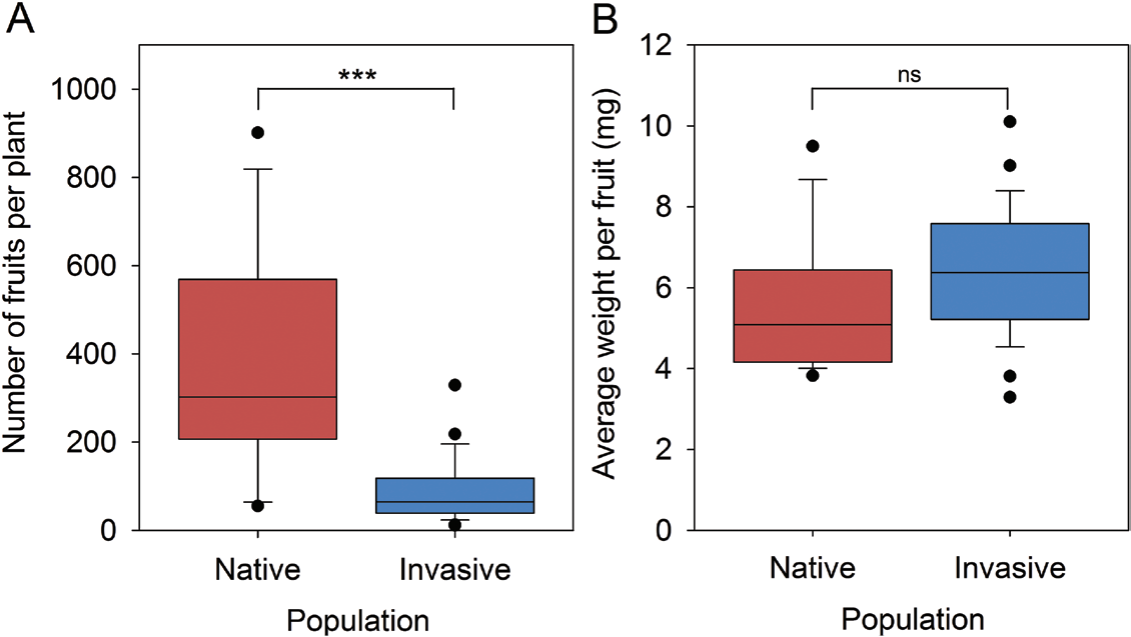
Reproductive output. (A) Number of fruits produced per plant for the native population (red, *n*=15 plants, average: 381 fruits per plant), and the invasive population (blue, *n*=27 plants, average: 91 fruits per plant). (B) Average fruit weight for the native population (red, *n*=15, average: 5.5 mg) and the invasive population (blue, *n*=27 plants, average: 6.3 mg). Box plots show the first, second, and third quartiles (the box), the 10th and 90th percentiles (the whiskers), and outliers as individual dots. Mann–Whitney U-tests were performed to test for significant differences between groups (ns *P*>0.05, ***P<0.001; Bonferroni correction was applied with *m*=17 to the α of individual comparisons to obtain the indicated overall α values).

Plants with a shorter vegetative phase have less time to build up resource-gathering organs for the production of seeds, so early flowering can be expected to decrease the reproductive output. We asked whether this was the case for the plants of the studied invasive population. First of all, the early flowering invasive plants had a lower final plant height ($\bar{\text{X}}$_n_=16.7 cm, $\bar{\text{X}}$_i_=9.1 cm, *P*<0.0001), main stem basal diameter ($\bar{\text{X}}$_n_=6.7 mm, $\bar{\text{X}}$_i_=2.5 mm, *P*<0.0001), number of branches on the main stem ($\bar{\text{X}}$_n_=27, $\bar{\text{X}}$_i_=9, *P*<0.0001), root area ($\bar{\text{X}}$_n_=36.5 cm^2^, $\bar{\text{X}}$_i_=9.4 cm^2^, *P*=0.0002), and root weight ($\bar{\text{X}}$_n_=905 mg, $\bar{\text{X}}$_i_=189 mg, *P*<0.0001) compared with the native plants (Supplementary Fig. S2). Consequently, the invasive plants produced 75% fewer fruits than native plants (*P*<0.0001; Fig. 2). This agrees with previous correlations of plant size with seed production (Dickerson and Sweet, 1971; Fumanal *et al.*, 2007). Although average fruit weight was slightly higher for plants from the invasive population, this difference was not significant (*P*=0.07). These results indicate that accelerated flowering in the invasive population is associated with a reproductive cost.

### *A. artemisiifolia* expresses three *FT/TFL1*-like genes

**Fig. 3.**
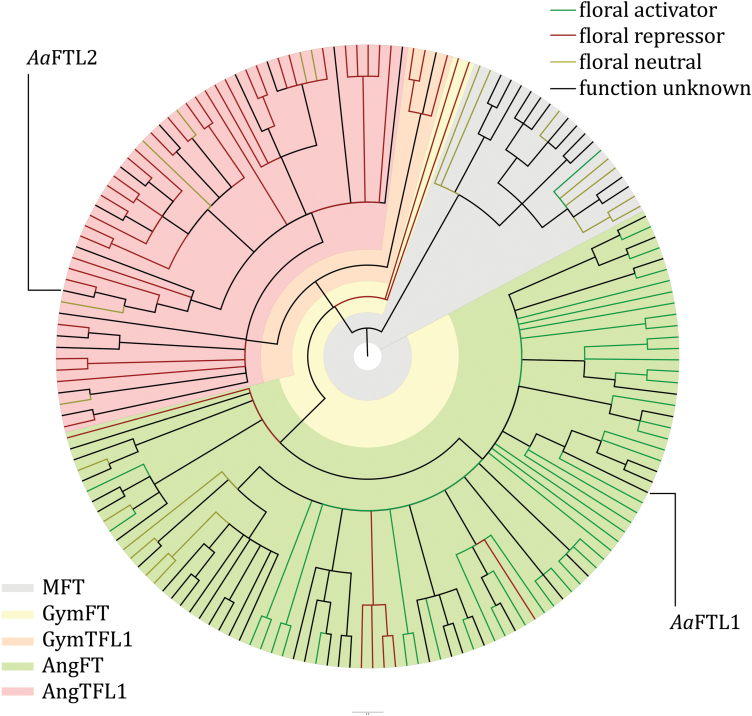
Phylogenetic tree of plant PEBPs. Cladogram of 172 PEBP sequences from 33 species belonging to 16 families of plants, inferred using a maximum likelihood method (Phyml); branches with <85% support were collapsed. Shading indicates the major clades: MFT (grey), gymnosperm FT (yellow), gymnosperm TFL1 (orange), angiosperm FT (green), angiosperm TFL1 (red). Branch colour indicates floral regulator potential: floral activators are green, floral neutral proteins are yellow, floral repressors are red, and proteins with unknown function in relation to flowering time are coloured black. The positions of the *A. artemisiifolia Aa*FTL1 and *Aa*FTL2 are indicated.

The transition to flowering is usually initiated by changes in gene expression. Here, we asked whether the observed differences in flowering time in invasive and native *A. artemisiifolia* populations are also associated with differential gene expression. Because of its important role in flowering time control, we focused on the *FT/TFL1* family of *A. artemisiifolia*. Little was known about *FT/TFL1* genes in *A. artemisiifolia* before, and we identified two members of this family using *A. artemisiifolia* and *A. trifida* EST libraries (Lai *et al.*, 2012). The partial sequence of a third member was reported by another laboratory (Li *et al.*, 2015). The partial sequences of *A. artemisiifolia FTL1* and *FTL2* were completed using 5'- and 3'-RACE. Full-length *AaFTL3* could not be obtained, presumably due to its low expression. However, the available sequence implies that *AaFTL3* is either dysfunctional or has a radically different function (see Supplementary Table S2 for the cDNA sequences of the three *A. artemisiifolia FTL* genes including GenBank accession numbers). *Ambrosia artemisiifolia FTL1* and *FTL2* were translated *in silico*, aligned with FTL protein sequences from other species, and used in a phylogenetic analysis. The resulting phylogenetic tree (Fig. 3) distinguishes the MFT clade (grey) from its FT and TFL1 daughter clades. The gymnosperm FT clade (yellow) and gymnosperm TFL1 (orange) can be distinguished from the angiosperm FT clade (green) and the angiosperm TFL1 clade (red) (see Supplementary Fig. S3 for a linear version of the same tree with all proteins labelled with name and accession number). *Ambrosia artemisiifolia* FTL1 belongs to the (angiosperm) FT clade and FTL2 to the (angiosperm) TFL1 clade. *Aa*FTL1 clusters together with the floral activator *H. annuus* FT4 (Blackman *et al.*, 2010) (*H. annuus* is the closest relative of *A. artemisiifolia* in the phylogenetic analysis, belonging to the same tribe Heliantheae within the Asteraceae family) and four *Chrysanthemum* sp. proteins (also from the Asteraceae family), two of which are floral activators (Oda *et al.*, 2012; Fu *et al.*, 2014) and the other two without known function. This clustering with floral activators suggests that *Aa*FTL1 could also be a floral activator. The second identified *A. artemisiifolia* FTL, *Aa*FTL2, clusters together with the floral repressor *C. seticuspe* AFT (Higuchi *et al.*, 2013) and *H. annuus* BROTHER OF FT AND TFL1 (BFT) (with unknown function), suggesting floral repressor activity. Another phylogenetic analysis (minimum evolution) confirmed the aforementioned clustering of *Aa*FTL1 and *Aa*FTL2 (Supplementary Fig. S4).

### Sequence composition predicts that *A. artemisiifolia* expresses one activator and one repressor of flowering

According to the phylogenetic analysis, the original angiosperm FT/TFL1 proteins presumably had a floral repressor function, since the gymnosperm FT/TFL1 proteins are floral repressors (Karlgren *et al.*, 2011; Liu *et al.*, 2016). Because of the ubiquitous presence of floral activators in the FT clade, it is reasonable to assume that the early angiosperm FT became a floral activator, and that later reversions occurred in some lineages. Because many mutations can cause loss of function, it should not be surprising that this seemed to have occurred multiple times in both the FT and TFL1 clade. To predict more precisely the function of the *A. artemisiifolia* FT/TFL1 proteins, a closer study of the amino acid sequences was required. Previous studies have identified critical functional regions of the FT/TFL1 proteins (Hanzawa *et al.*, 2005; Ahn *et al.*, 2006; Danilevskaya *et al.*, 2008). Most strikingly, a single amino acid residue can make the difference between a floral activator and a floral repressor (Hanzawa *et al.*, 2005). The floral activating function of Arabidopsis FT and the repressive function of TFL1 can be interchanged by swapping a histidine residue (at position 88 for Arabidopsis TFL1) for a tyrosine residue (at position 85 for Arabidopsis FT) (Hanzawa *et al.*, 2005). *Aa*FTL1 possesses the tyrosine residue that confers the activating function to Arabidopsis FT (Fig. S5) and *Aa*FTL2 possesses the histidine residue that confers repressive function to Arabidopsis TFL1 (Supplementary Fig. S6), supporting the notion that *Aa*FTL1 is a floral activator and *Aa*FTL2 a floral repressor.

For Arabidopsis FT and TFL1, this residue is important for their function in the regulation of flowering, but it is not the only position that determines whether a FT/TFL1 protein functions like a floral activator or repressor. This is exemplified by Arabidopsis BFT, which has floral inhibitory activity, but possesses the ‘activator’ tyrosine residue that Arabidopsis FT has. To predict protein functionality more accurately, a sequence analysis was performed to determine what other amino acid residues can distinguish a floral activator from a floral repressor. This analysis was performed within either the FT-clade (for *Aa*FTL1) or the TFL1-clade (for *Aa*FTL2), to avoid creating a bias for positions that reflect evolutionary history rather than function. Within the FT-clade, there are activators, repressors, and flowering neutral proteins, and a consensus sequence was made of each of these three groups. The same procedure was followed within the TFL1-clade. For each consensus sequence, positions unique to that consensus (e.g. positions in the FT-activator consensus that do not occur in the FT-repressor consensus or FT-neutral consensus) were selected. Then, the *A. artemisiifolia* sequences were analysed at the selected positions. Within the FT-clade, *Aa*FTL1 possesses a higher proportion of residues unique to the consensus activator (10/13), than to the neutral consensus (2/16) or to the consensus repressor (1/18) (Supplementary Fig. S5). *Aa*FTL2 possesses two residues unique to repressors (2/9) and none unique to the neutral consensus (0/1) (Supplementary Fig. S6). In short, this analysis also indicates that *Aa*FTL1 is a floral activator and *Aa*FTL2 a floral repressor.

As an independent test for likely *Aa*FTL1 and *Aa*FTL2 function, we calculated scores that provide quantitative measures of sequence similarity for a test sequence to a group of proteins with shared function (see the Materials and methods for details). Unlike using consensus sequences, this analysis retains amino acid residues that are present in >50% of the sequences. For proteins in the FT-clade, the average activator ratio (i.e. similarity to flowering activators divided by similarity to non-activators) is significantly different between activator and non-activator groups ($\bar{\text{X}}$_act_=1.16, $\bar{\text{X}}$_non_=1.01, *P*<0.001), showing the usefulness of this score for the prediction of FT/TFL1 function (Supplementary Fig. S7A). *Aa*FTL1 has a ratio of 1.18, which is similar to the average for known activators and well outside of the non-activator distribution, indicating that *Aa*FTL1 has a floral activator function (Supplementary Fig. S7A). For TFL1-like proteins, the two functional groups have significantly different average repressor ratios ($\bar{\text{X}}$_rep_=1.00, $\bar{\text{X}}$_non_=0.97, *P*<0.001) (Supplementary Fig. S7B). *Aa*FTL2 has a repressor ratio of 0.99, which is slightly lower than the average for repressors but higher than any non-repressor. Using an empirically determined cut-off to call as many proteins correctly as possible (0.98, calling only one true repressor falsely a non-repressor), *Aa*FTL2 is called as a repressor, as is the closely related *Ha*BFT. Together, the used sequence analysis methods consistently predict that *Aa*FTL1 is a floral activator and *Aa*FTL2 a floral repressor.

**Fig. 4.**
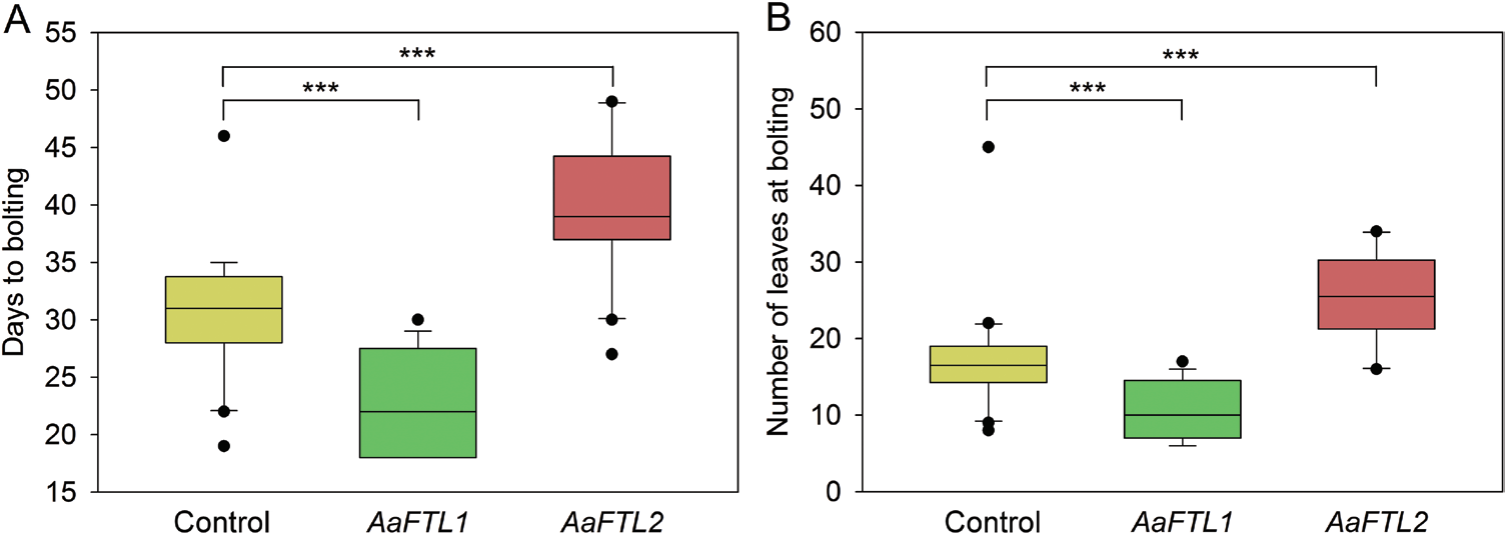
Transgenic expression of *AaFTL1* and *AaFTL2* in Arabidopsis. Box plots of the duration of the vegetative phase in days to bolting (A) and number of rosette leaves at bolting (B) of T_1_ plants containing pMDC160 (control, yellow), and of plants expressing the coding sequences of *AaFTL1* (green) or *AaFTL2* (red) under the constitutive 35S CaMV promoter. Box plots show the first, second, and third quartiles (the box), the 10th and 90th percentiles (the whiskers), and outliers as individual dots. Mann–Whitney U-tests were performed to test for significant differences between groups (***P<0.001; Bonferroni correction was applied with *m*=4 to the α of individual comparisons to obtain the indicated overall α values).

To establish experimental proof for *Aa*FTL1’s ability to promote flowering and for *Aa*FTL2’s ability to repress flowering, we transformed *35S:AaFTL1*, *35S:AaFTL2*, and empty vector controls into Arabidopsis. The expression of the constructs was confirmed by RT–qPCR (Supplementary Fig. S8A). In addition, the expression of two direct targets of Arabidopsis FT, *SOC1* and *FUL*, was measured (Supplementary Fig. S8B). We observed that *SOC1* expression was increased in both overexpressors, and that *FUL* expression was down-regulated in the *AaFTL2* overexpression line. This was unexpected as we hypothesized that FTL1 and FTL2 would simply have opposite effects on FT targets. Part of the explanation may be the heterologous expression system together with the evolutionary divergence between Arabidopsis and *A. artemisiifolia*, or the use of the strong ectopic 35S promoter. Nevertheless, as SOC1 and FUL form heterodimers to regulate the floral transition (Balanzà *et al.*, 2014), the observed expression changes predict accelerated flowering of *Aa*FTL1 lines and delayed flowering of *Aa*FTL2 lines. These predictions are consistent with the observed flowering time. *AaFTL1*-expressing plants flowered earlier than control plants, on average after 23 d instead of 31 d (*P*<0.0001), forming fewer leaves (11 instead of 17 leaves, *P*<0.0001; Fig. 4). *AaFTL2*-expressing plants flowered significantly later than the control plants, on average after 39 d (*P*<0.0001), forming more leaves (25 leaves, *P*<0.0001, Fig. 4). Together, functional tests and sequence-based predictions firmly established that *Aa*FTL1 and *Aa*FTL2 are floral activators and repressors, respectively.

### *FT/TFL1* gene expression is associated with flowering time in *A. artemisiifolia*

**Fig. 5.**
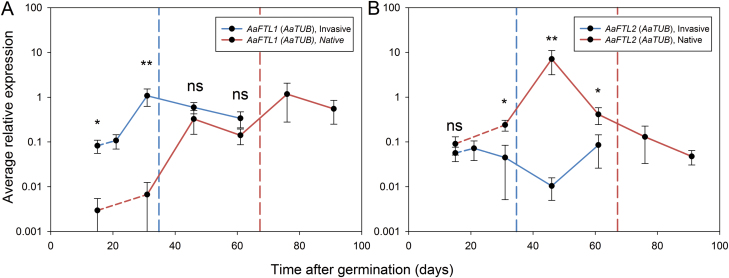
Expression of *AaFTL1* and *AaFTL2*. Data points indicate average expression values for *AaFTL1* (A) and *AaFTL2* (B) of plants from the invasive (blue) and native population (red), respectively. The expression shown here is based on four (native) or 10 (invasive) biological replicates, each with three technical replicates. The data for the 15 DAG time point were obtained in a separate experiment with four pools of four plants each as biological replicates. Error bars show the SE. *AaTUB* was used as a reference gene. Mann–Whitney U-tests were performed to test for significant differences between populations (ns *P*>0.05, *P<0.05, **P<0.01). The vertical dashed lines indicate the average male floral initiation time for the invasive (blue) and the native population (red), respectively.

Having confirmed the functionality of *Aa*FTL1 as a floral activator and of *Aa*FTL2 as a floral repressor, the expression of these *A. artemisiifolia FT/TFL1* genes was investigated to determine whether interpopulation differences in expression level correlate with the differences in flowering time. The *AaFTL1* and *AaFTL2* transcript levels were determined at several different time points in *A. artemisiifolia* plants (Fig. 5; Supplementary Fig. S9). At the first time point measured (15 DAG), the invasive population already had higher transcript levels of the floral activator *AaFTL1* than the native population (*P*=0.029; Fig. 5). The expression further increased at 31 DAG in the invasive population, but not in the native population (*P*=0.006). Only at 46 DAG did *AaFTL1* expression levels in the native population reach the levels in the invasive population without much further change until 61 DAG (p_46DAG_=0.44, p_61DAG_=0.52). *AaFTL2* expression was initially similar in the two populations (*P*=0.686), but increased in the native population at 31 DAG and stayed higher until at least 61 DAG (p_31DAG_=0.028, p_46DAG_=0.006, p_61DAG_=0.028). The third *FT/TFL1* gene was also investigated: its expression level was similarly low in both populations (see Supplementary Fig. S9). The subset of plants from both populations used for the expression analysis did not significantly differ in phenological characteristics from the larger populations used to generate Fig. 1 (Supplementary Fig. S10), indicating that the gene expression results are representative for the whole population. Repressive FT/TFL1-like proteins can have anti-florigenic function, being expressed in leaves and moving to the shoot apex to repress flowering (Foucher *et al.*, 2003; Harig *et al.*, 2012; Higuchi *et al.*, 2013), but they can also be mostly maintainers of meristem indeterminacy, expressed at specific regions in the shoot apex to prevent conversion of the shoot apical meristem to a floral meristem (Shannon and Meeks-Wagner, 1991; Bradley *et al.*, 1996; Foucher *et al.*, 2003). *AaFTL1* and *AaFTL2* expression was therefore compared between *A. artemisiifolia* leaves and shoot apices. The results established that both genes are expressed in both leaves and shoot apices, but that expression levels of both genes were several orders of magnitude higher in leaves than in apices (Supplementary Fig. S11). This result suggests that *Aa*FTL1 may represent canonical florigen function in *A. artemisiifolia* while *Aa*FTL2 may be an anti-florigen similar to the closely related *Cs*AFT instead of a meristematic maintainer of indeterminacy.

## Discussion

Common ragweed plants introduced to Germany flower earlier than plants from the native range. The invasive plants have a shorter vegetative phase, while the male maturation phase remains unchanged. This early flowering trait comes with a cost in reproductive output. Plants from the invasive population produce 75% less fruits, and consequently less seeds, but without showing a significant increase in the size of the individual fruit. Because fruit development in *A. artemisiifolia* is frost sensitive, reduced fruit set of early flowering plants is a fitness disadvantage only in places with long vegetation periods such as the original range of the native population (Supplementary Fig. S12) and in invaded southern Europe. In contrast, under short vegetation periods, only early-flowering plants can produce any viable seeds, making the higher seed set of late-flowering plants irrelevant. In our study, the invasive plants flowered ~30 d earlier than the native plants, and one might wonder whether this difference is great enough to produce seeds successfully where the native population cannot. Our study was conducted under flowering-inducing SD conditions at optimal growth temperatures, showing the earliest times of flowering in the two populations. In the field, this difference becomes more pronounced at higher latitudes, presumably due to longer day lengths in the summer time (triggering flowering later), and lower temperatures (extending all phenological phases due to slower growth). The difference is ~30 d at Osijek (latitude N59°48'55'') and ~70 d in Uppsala (latitude N45°31'16'') (Scalone *et al.*, 2016). After pollination, seeds can take 4–6 weeks to develop (Essl *et al.*, 2015), which means that by early flowering, invasive plants could complete seed development in the common garden before the native plants have even started to release pollen. This indicates that indeed early flowering could mean the difference between some progeny or no progeny at all.

Plants from the studied invasive population not only were early flowering but also had increased dichogamy. It is thought that the self-incompatibility of *A. artemisiifolia* is leaky (Friedman and Barrett, 2008; Essl *et al.*, 2015) and the architecture of the inflorescences and release of pollen onto lower tissue guarantee that pistils will encounter self-pollen, so additional blocks preventing inbreeding may be adaptive. For invasive species, however, it is thought that selfing is beneficial as populations are initially small and mating partners are rare (Baker, 1955, 1974). In fact, male and female mature phases still overlap considerably in invasive *A. artemisiifolia*, and effects on selfing efficiency may thus be limited. It is possible that increased dichogamy in invasive *A. artemisiifolia* is a by-product of earlier flowering rather than an independent adaptation.

Crossings of plants from both populations showed that the alleles underlying the shortening of the vegetative phase and of the male maturation phase are dominant, which (when adaptive) generally spread faster in a population than recessive alleles. The common garden experiment (Scalone *et al.*, 2016) indicated that the shorter vegetative phase already allows for stable populations of invasive *A. artemisiifolia* north of the current distribution. Distribution modelling using phenological data of this invasive population confirms that this trait allows *A. artemisiifolia* to expand north without the need for additional climate change (Chapman *et al.*, 2017). In addition, the shorter male maturation phase of F_1_ plants indicates that the gene pool allows for additional phenological changes that potentially lead to adaptation to even higher latitudes.

The early-flowering trait of the analysed invasive *A. artemisiifolia* plants is accompanied by an earlier activation of a floral activator and lower expression of a floral repressor. It is not certain whether both these differences contribute to the difference in flowering time, but it is plausible: at 46 DAG, the activator *AaFTL1* is expressed equally highly in plants of both populations, although plants from the native population flower only 3 weeks later (on average at 67 DAG). This delay may be caused by the transient increase of the expression of the predicted floral repressor *AaFTL2* at ~46 DAG, preventing floral initiation by *AaFTL1*. At 61 DAG, the level of the repressor has decreased again, allowing for flowering even in the native population. Thus, the observed differences in expression of *FT/TFL1* genes can explain the differences in flowering time between the native and the invasive *A. artemisiifolia* populations, and there is no evidence suggesting additional differences in *FT/TFL1*-independent pathways.

The expression of the two *A. artemisiifolia FTL* genes differs in three ways between the two populations: the invasive population has (i) a higher baseline expression of the floral activator *AaFTL1* as well as (ii) an earlier up-regulation of that gene, and (iii) shows no considerable induction of the floral repressor *AaFTL2*. The causal mutation(s) for differences in flowering time are likely to affect both *AaFTL1* and *AaFTL2* expression but, because flowering was accelerated under both LD and SD conditions, it is likely that photoperiod-independent input to the *FT/TFL1* floral integrators is altered in the invasive *A. artemisiifolia* population. Because of the large number of possible candidates, genetic mapping will be more appropriate than candidate gene approaches to identify causal mutations for early flowering in the invasive *A. artemisiifolia* population.

We noticed that the native population had larger variability in flowering time (time to male floral initiation) than the invasive population (SE of 4.6 and 0.9 d, respectively). The earliest plant from the native population flowered at 36 DAG, right in the middle of the distribution of flowering time for plants from the invasive population. This indicates that the alleles conferring early flowering in the studied invasive population could have pre-existed in the ancestral native population. Indeed, flowering time varies among different populations in the northern part of the USA, and there is an unidentified genetic component underlying this variation (Stinson *et al.*, 2016). The invasive population is from a location similar in terms of the length of the growth period (when temperatures are >5 °C) to the northernmost border of the *A. artemisiifolia* distribution in America, for instance in Toronto (Supplementary Fig. S12). Wind dispersal of pollen can facilitate exchange of alleles between distant *A. artemisiifolia* populations, introducing alleles for early flowering even to more southern populations, from which the European populations may have originated. Future studies on the nature and distribution of alleles causing early flowering in *A. artemisiifolia* together with genome-wide scans for signs of selective sweeps will establish what impact pre-existing alleles had on the invasive success of *A. artemisiifolia*. Despite the aforementioned arguments for the change of flowering time as a common method of adaptation, it may be warranted to study other invasive *A. artemisiifolia* populations to determine how widespread this mechanism is.

## Supplementary data

Supplementary data are available at *JXB* online.

Fig. S1. Overview of *A. artemisiifolia* phenology.

Fig. S2. Final plant characteristics.

Fig. S3. Linear version of the phylogenetic tree shown in Fig. 3.

Fig. S4. Phylogenetic tree based on the minimum evolution method.

Fig. S5. Alignment of functional consensus sequences and *A. artemisiifolia* FTL1.

Fig. S6. Alignment of functional consensus sequences and *A. artemisiifolia* FTL2.

Fig. S7. Activator and repressor ratios.

Fig. S8. Gene expression in transgenic Arabidopsis plants expressing *AaFTL1* and *AaFTL2*.

Fig. S9. Average relative expression of *A. artemisiifolia AaFTL1*, *AaFTL2*, and *AaFTL3*.

Fig. S10. Comparison of flowering phenology of total and sample populations.

Fig. S11. *AaFTL1* and *AaFTL2* expression in leaves and shoot apices.

Fig. S12. Day length and vegetation period at the localities of the two studied *A. artemisiifolia* populations.

Table S1. Primers used in this study.

Table S2. *A. artemisiifolia FT/TFL1* cDNA sequences.

Table S3. List of FT-like proteins with known and predicted function.

Table S4. List of TFL1-like proteins with known and predicted function.

Table S5. Standard errors of gene expression values for different reference genes as established by RT–qPCR.

ery100_suppl_Supplementary_Figures_and_Tables
