# Supplementary material for: North European invasion by common ragweed is associated with early flowering and dominant changes in FT/TFL1 expression
Source: J Exp Bot. 2018 Mar 14;69(10):2647–58. doi: 10.1093/jxb/ery100 (PMC5920306; doi:10.1093/jxb/ery100)
Supplement: ery100_suppl_Supplementary_Figures_and_Tables [file ery100_suppl_supplementary_figures_and_tables.pdf]

## Supplementary Figures

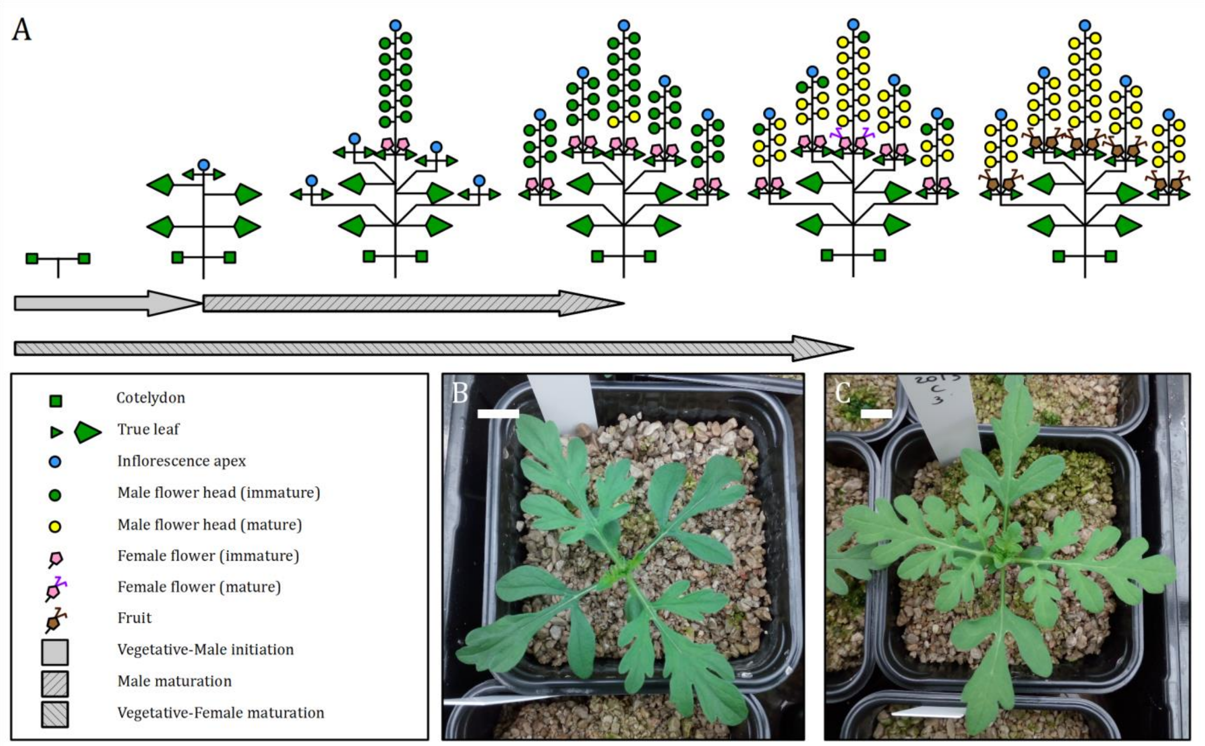


**Figure S1. Overview of *Ambrosia artemisiifolia* phenology**. (A) Schematic overview of *A. artemisiifolia* phenology from germinated seedling to a plant with mature fruit. Arrows indicate the phases that have been measured and displayed in Fig. 1. The "Vegetative-Male initiation" phase is from germination to the appearance of the main (male) inflorescence bud. The "Male maturation" phase starts from the appearance of the main inflorescence bud and ends with the appearance of the first mature male flowers (designated by the release of pollen). The "Vegetative-Female maturation" phase starts from germination and lasts until the appearance of the first mature female flower (designated by the extension of dichotomous stigmas). (B) Image of a plant from the native population at the pre-flowering stage 15 DAG. (C) Image of a plant from the invasive population at the pre-flowering stage 15 DAG. Scale bars = 1cm.

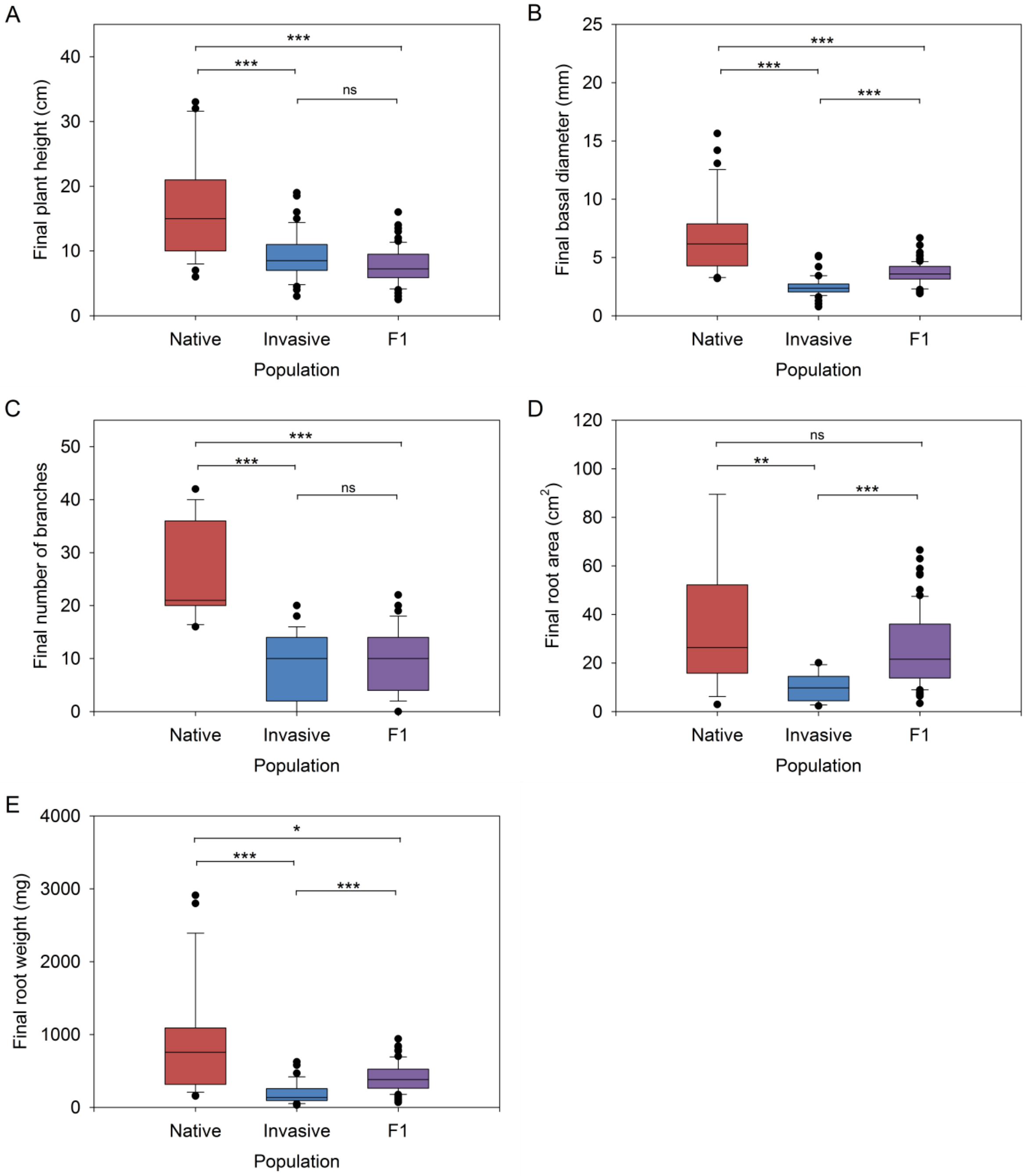


**Figure S2. Final plant characteristics.** Characteristics shown are final plant height in cm (A), final basal diameter in mm (B), final number of branches (C), final root area in cm² (D) and final root weight in mg (E) for the native population (red), invasive population (blue), and the F1 population (purple). Box plots show the first, second and third quartiles (the box), the 10th and 90th percentiles (the whiskers), and outliers as individual dots. Mann-Whitney U tests were performed to check for significant differences between groups (ns p>0.05, * p<0.05, ** p<0.01, *** p<0.001, Bonferroni correction was applied with m=17 to the α of individual comparisons to obtain the indicated overall α values).

**Figure S3. Linear version of the phylogenetic tree shown in Fig. 3 (on the next page).** Labels indicate accession code, name, and species. Line and text colour indicate known potential function (floral activator=green, floral repressor=red, flowering neutral=yellow). Shading indicates major clades (grey=MFT, yellow= gymnosperm FT, orange= gymnosperm TFL1 green=angiosperm FT, red= angiosperm TFL1).

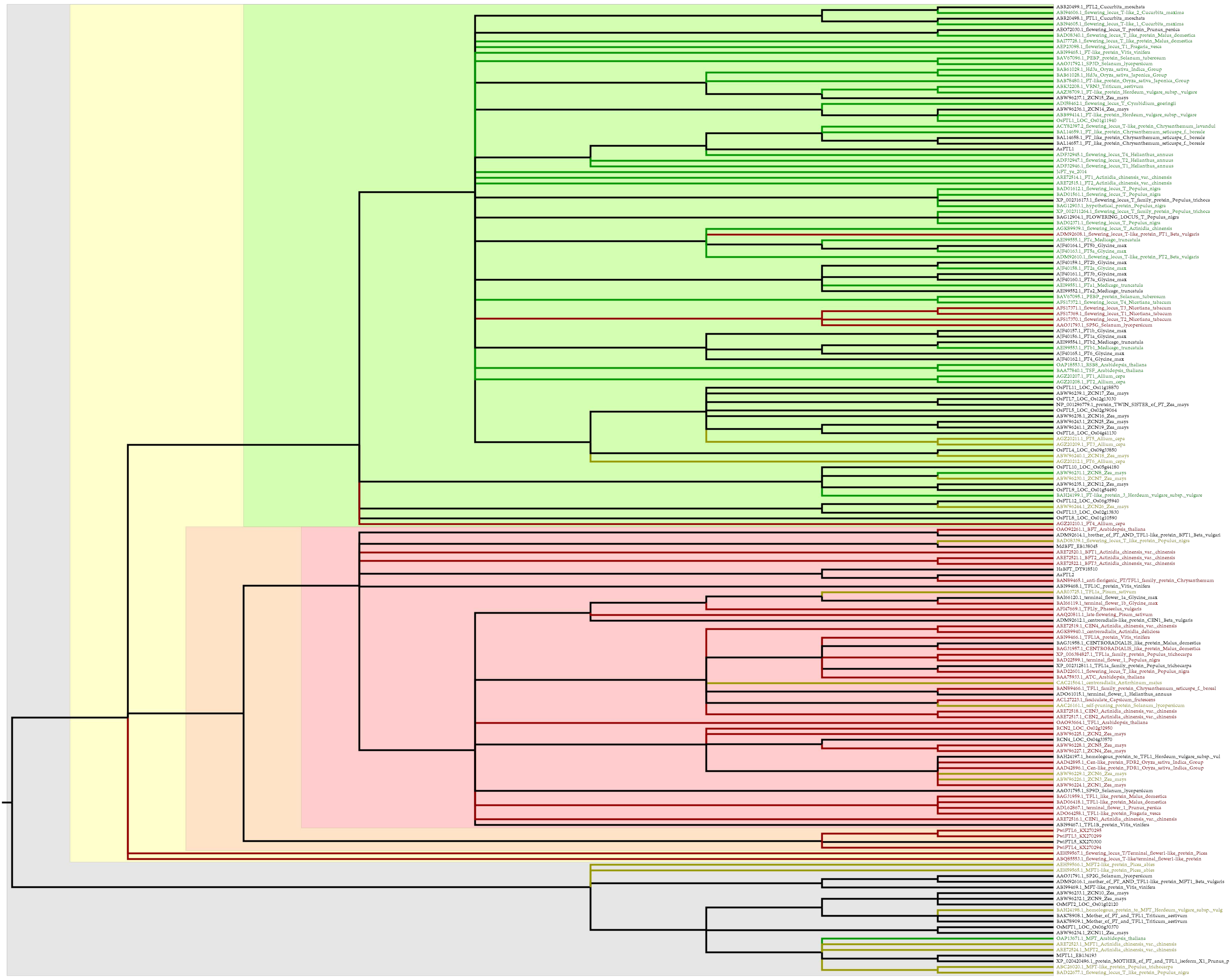
ABR20499.1_FTL2_Cucurbita_moschata
ABI94606.1_flowering_locus_T-like_2_Cucurbita_maxima
ABR20498.1_FTL1_Cucurbita_moschata
ABI94605.1_flowering_locus_T-like_1_Cucurbita_maxima
AEO72030.1_flowering_locus_T_protein_Prunus_persica
BAD08340.1_flowering_locus_T_like_protein_Malus_domestica
BAI77728.1_flowering_locus_T_like_protein_Malus_domestica
AEP23098.1_flowering_locus_T1_Fragaria_vesca
ABI99465.1_FT-like_protein_Vitis_vinifera
BAV67096.1_PEBP_protein_Solanum_tuberosum
AAO31792.1_SP3D_Solanum_lycopersicum
BAB61029.1_Hd3a_Oryza_sativa_Indica_Group
BAB61028.1_Hd3a_Oryza_sativa_Japonica_Group
BAB78480.1_FT-like_protein_Oryza_sativa_Japonica_Group
ABK32208.1_VRN3_Triticum_aestivum
AAZ38709.1_FT-like_protein_Hordeum_vulgare_subsp._vulgare
ABW96237.1_ZCN15_Zea_mays
ADI58462.1_flowering_locus_T_Cymbidium_goeringii
ABW96236.1_ZCN14_Zea_mays
ABB99414.1_FT-like_protein_Hordeum_vulgare_subsp._vulgare
OsFTL1_LOC_Os01g11940
ACY82397.2_flowering_locus_T-like_protein_Chrysanthemum_lavandul
BAL14659.1_FT_like_protein_Chrysanthemum_seticuspe_f._boreale
BAL14658.1_FT_like_protein_Chrysanthemum_seticuspe_f._boreale
BAL14657.1_FT_like_protein_Chrysanthemum_seticuspe_f._boreale
AaFTL1
ADF32945.1_flowering_locus_T4_Helianthus_annuus
ADF32947.1_flowering_locus_T2_Helianthus_annuus
ADF32946.1_flowering_locus_T1_Helianthus_annuus
JcFT_ye_2014
ARE72514.1_FT1_Actinidia_chinensis_var._chinensis
ARE72515.1_FT2_Actinidia_chinensis_var._chinensis
BAD01612.1_flowering_locus_T_Populus_nigra
BAD01561.1_flowering_locus_T_Populus_nigra
XP_002316173.1_flowering_locus_T_family_protein_Populus_trichoca
BAG12903.1_hypothetical_protein_Populus_nigra
XP_002311264.1_flowering_locus_T_family_protein_Populus_trichoca
BAG12904.1_FLOWERING_LOCUS_T_Populus_nigra
BAD02371.1_flowering_locus_T_Populus_nigra
AGK89939.1_flowering_locus_T_Actinidia_chinensis
ADM92608.1_flowering_locus_T-like_protein_FT1_Beta_vulgaris
AEI99555.1_FTc_Medicago_truncatula
AJF40164.1_FT5b_Glycine_max
AJF40163.1_FT5a_Glycine_max
ADM92610.1_flowering_locus_T-like_protein_FT2_Beta_vulgaris
AJF40159.1_FT2b_Glycine_max
AJF40158.1_FT2a_Glycine_max
AJF40161.1_FT3b_Glycine_max
AJF40160.1_FT3a_Glycine_max
AEI99551.1_FTa1_Medicago_truncatula
AEI99552.1_FTa2_Medicago_truncatula
BAV67095.1_PEBP_protein_Solanum_tuberosum
AFS17372.1_flowering_locus_T4_Nicotiana_tabacum
AFS17371.1_flowering_locus_T3_Nicotiana_tabacum
AFS17369.1_flowering_locus_T1_Nicotiana_tabacum
AFS17370.1_flowering_locus_T2_Nicotiana_tabacum
AAO31793.1_SP5G_Solanum_lycopersicum
AJF40157.1_FT1b_Glycine_max
AJF40156.1_FT1a_Glycine_max
AEI99554.1_FTb2_Medicago_truncatula
AEI99553.1_FTb1_Medicago_truncatula
AJF40165.1_FT6_Glycine_max
AJF40162.1_FT4_Glycine_max
OAP18553.1_RSB8_Arabidopsis_thaliana
BAA77840.1_TSF_Arabidopsis_thaliana
AGZ20207.1_FT1_Allium_cepa
AGZ20208.1_FT2_Allium_cepa
OsFTL11_LOC_Os11g18870
ABW96239.1_ZCN17_Zea_mays
OsFTL7_LOC_Os12g13030
NP_001296779.1_protein_TWIN_SISTER_of_FT_Zea_mays
OsFTL5_LOC_Os02g39064
ABW96238.1_ZCN16_Zea_mays
ABW96243.1_ZCN25_Zea_mays
ABW96241.1_ZCN19_Zea_mays
OsFTL6_LOC_Os04g41130
AGZ20211.1_FT5_Allium_cepa
AGZ20209.1_FT3_Allium_cepa
OsFTL4_LOC_Os09g33850
ABW96240.1_ZCN18_Zea_mays
AGZ20212.1_FT6_Allium_cepa
OsFTL10_LOC_Os05g44180
ABW96231.1_ZCN8_Zea_mays
ABW96230.1_ZCN7_Zea_mays
ABW96235.1_ZCN12_Zea_mays
OsFTL9_LOC_Os01g54490
BAH24199.1_FT-like_protein_3_Hordeum_vulgare_subsp._vulgare
OsFTL12_LOC_Os06g35940
ABW96244.1_ZCN26_Zea_mays
OsFTL13_LOC_Os02g13830
OsFTL8_LOC_Os01g10590
AGZ20210.1_FT4_Allium_cepa
OAO92261.1_BFT_Arabidopsis_thaliana
ADM92614.1_brother_of_FT_AND_TFL1-like_protein_BFT1_Beta_vulgari
BAD08339.1_flowering_locus_T_like_protein_Populus_nigra
MdBFT_EB138045
ARE72520.1_BFT1_Actinidia_chinensis_var._chinensis
ARE72521.1_BFT2_Actinidia_chinensis_var._chinensis
ARE72522.1_BFT3_Actinidia_chinensis_var._chinensis
HaBFT_DY918510
AaFTL2
BAN89465.1_anti-florigenic_FT/TFL1_family_protein_Chrysanthemum
ABI99468.1_TFL1C_protein_Vitis_vinifera
AAR03725.1_TFL1a_Pisum_sativum
BAI66120.1_terminal_flower_1a_Glycine_max
BAI66119.1_terminal_flower_1b_Glycine_max
AFI47669.1_TFLly_Phaseolus_vulgaris
AAQ20811.1_late-flowering_Pisum_sativum
ADM92612.1_centroradialis-like_protein_CEN1_Beta_vulgaris
ARE72519.1_CEN4_Actinidia_chinensis_var._chinensis
AGK89940.1_centroradialis_Actinidia_deliciosa
ABI99466.1_TFL1A_protein_Vitis_vinifera
BAG31958.1_CENTRORADIALIS_like_protein_Malus_domestica
BAG31957.1_CENTRORADIALIS_like_protein_Malus_domestica
XP_006384827.1_TFL1a_family_protein_Populus_trichocarpa
BAD22599.1_terminal_flower_1_Populus_nigra
XP_002312811.1_TFL1a_family_protein_Populus_trichocarpa
BAD22601.1_flowering_locus_T_like_protein_Populus_nigra
BAA75933.1_ATC_Arabidopsis_thaliana
CAC21564.1_centroradialis_Antirrhinum_majus
BAN89466.1_TFL1_family_protein_Chrysanthemum_seticuspe_f._boreal
ADO61015.1_terminal_flower_1_Helianthus_annuus
ACL27223.1_fasciculate_Capsicum_frutescens
AAC26161.1_self-pruning_protein_Solanum_lycopersicum
ARE72518.1_CEN3_Actinidia_chinensis_var._chinensis
ARE72517.1_CEN2_Actinidia_chinensis_var._chinensis
OAO93664.1_TFL1_Arabidopsis_thaliana
RCN2_LOC_Os02g32950
ABW96225.1_ZCN2_Zea_mays
RCN4_LOC_Os04g33570
ABW96228.1_ZCN5_Zea_mays
ABW96227.1_ZCN4_Zea_mays
BAH24197.1_homologous_protein_to_TFL1_Hordeum_vulgare_subsp._vul
AAD42895.1_Cen-like_protein_FDR2_Oryza_sativa_Indica_Group
AAD42896.1_Cen-like_protein_FDR1_Oryza_sativa_Indica_Group
ABW96229.1_ZCN6_Zea_mays
ABW96226.1_ZCN3_Zea_mays
ABW96224.1_ZCN1_Zea_mays
AAO31795.1_SP9D_Solanum_lycopersicum
BAG31959.1_TFL1_like_protein_Malus_domestica
BAD06418.1_TFL1-like_protein_Malus_domestica
ADL62867.1_terminal_flower_1_Prunus_persica
ADO64258.1_TFL1-like_protein_Fragaria_vesca
ARE72516.1_CEN1_Actinidia_chinensis_var._chinensis
ABI99467.1_TFL1B_protein_Vitis_vinifera
PwiFTL6_KX270295
PwiFTL3_KX270299
PwiFTL5_KX270300
PwiFTL4_KX270294
AEH59567.1_flowering_locus_T/Terminal_flower1-like_protein_Picea
ABQ85553.1_flowering_locus_T-like/terminal_flower1-like_protein
AEH59566.1_MFT2-like_protein_Picea_abies
AEH59565.1_MFT1-like_protein_Picea_abies
AAO31791.1_SP2G_Solanum_lycopersicum
ADM92616.1_mother_of_FT_AND_TFL1-like_protein_MFT1_Beta_vulgaris
ABI99469.1_MFT-like_protein_Vitis_vinifera
ABW96233.1_ZCN10_Zea_mays
ABW96232.1_ZCN9_Zea_mays
OsMFT2_LOC_Os01g02120
BAH24198.1_homologous_protein_to_MFT_Hordeum_vulgare_subsp._vulg
BAK78908.1_Mother_of_FT_and_TFL1_Triticum_aestivum
BAK78909.1_Mother_of_FT_and_TFL1_Triticum_aestivum
OsMFT1_LOC_Os06g30370
ABW96234.1_ZCN11_Zea_mays
OAP13671.1_MFT_Arabidopsis_thaliana
ARE72523.1_MFT1_Actinidia_chinensis_var._chinensis
ARE72524.1_MFT2_Actinidia_chinensis_var._chinensis
MFTL1_EB134193
XP_020420496.1_protein_MOTHER_of_FT_and_TFL1_isoform_X1_Prunus_p
ABC26020.1_MFT-like_protein_Populus_trichocarpa
BAD22677.1_flowering_locus_T_like_protein_Populus_nigra

**Figure S4. Phylogenetic tree based on the minimum evolution method (on the next page).** This is a consensus tree based on 100 bootstrap replicates. Labels indicate accession code, name, and species. Line and text colour indicate known potential function (floral activator=green, floral repressor=red, flowering neutral=yellow). Shading indicates major clades (grey=MFT, yellow=gymnosperm FT, orange= gymnosperm TFL1, green=angiosperm FT, red= angiosperm TFL1).

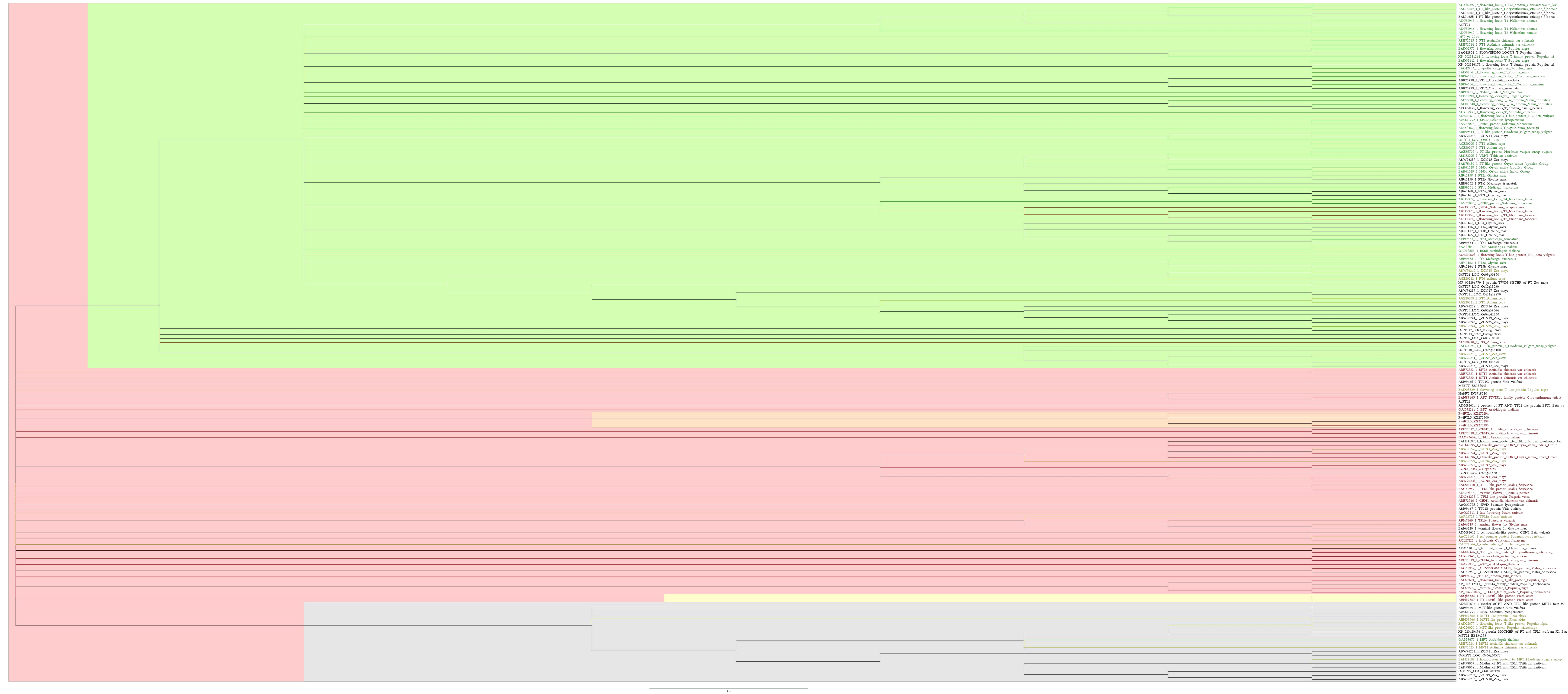

```
               ....|....| ....|....| ....|....| ....|....| ....|....|
                   5          15         25         35         45
FT_ACT         PLVVGRVIGD VLDPFTRSIS LRVTYPSQVV NQPRVDVMVD PDAPSPSDPN
FT_NEU         PLV~G~VIGD VLDPFTKSAS LR~TYPS~V~ N~PRVEVM~D PDAPSPSNPT
FT_REP         PL~VSGVIGD VLDPFTRS~D F~VVYPSQIV NQPRVDVMVD PDAPTPSNPN
AaFTL1         PLVVGRVIGD VLESFTKSMN LTVSYPSQVV NQPRVEVMVD PDAPSPSDPN

               ....|....| ....|....| ....|....| ....|....| ....|....|
                   55         65         75         85         95
FT_ACT         LREYLHWLVT DIPATTGASF GQEVVCYESP RPTMGIHRFV FVLFRQRQNF
FT_NEU         ~REYLHWMVT DIPE~~DASF GNEIVPYESP QPTAGIHR~V FVLFKQRQNF
FT_REP         LREYLHWLVT DIPATTGA~F GNEIV~YESP RPSLGIHRYI FVLFRQRQNF
AaFTL1         LREYLHWLVT DIPATTGARF GQEVVCYESP RPSMGIHRMV FVLFRQRQNF

               ....|....| ....|....| ....|....
                  105        115        125
FT_ACT         NTRDFAELYN LGSPVAAVYF NCQRESGSG
FT_NEU         NSRDFAAYYN LGPPVAAVYF NCQRE~GCG
FT_REP         NTRDFARFHN L~LPVAAVYF NCNREGGTG
AaFTL1         NTKDFAELYN LGSPVAAVYF NCQRESGFG
```

**Figure S5. Alignment of functional consensus sequences and *A. artemisiifolia* FTL1.** FT-clade activator consensus (FT_ACT), FT-clade neutral consensus (FT_NEU), FT-clade repressor consensus (FT_REP), and *Aa*FTL1. Consensus sequences are based on 45, 6, and 6 sequences, respectively. Green highlights indicate amino acid residues unique for the activator consensus, yellow highlights for residues unique to the neutral consensus, red for residues unique to the repressor consensus, grey for residues not matching any consensus, and in purple is the tyrosine residue that confers floral activating function to *Arabidopsis thaliana* FT. Tildes indicate positions without a predominant residue.

```
            ....|....| ....|....| ....|....| ....|....| ....|....|
                   5          15         25         35         45
TFL1_NEU    PLVVGRVIGE V~DSFTPSVK M~VTYPSAV~ SKPRVE~MTD PDVPGPSDPY
TFL1_REP    PLVVGRVIGD VLDSFTPSVK MTVTYPSAVT SKPRVEVMTD PDVPGPSDPY
AaFTL2      SLVVGRVIGD VVDQFTPSAQ MDVIYPNLVA SKPRVHVMTD PDAPSPSDPY

            ....|....| ....|....| ....|....| ....|....| ....|....|
                  55         65         75         85         95
TFL1_NEU    LREHLHWIVT DIPGTTDASF G~E~VSYE~P RPNIGIHRFV FVLFKQRDHF
TFL1_REP    LREHLHWIVT DIPGTTDASF GREVVSYEMP RPNIGIHRFV FVLFKQRDHF
AaFTL2      LREHLHWIVT DIPGTTDATF GREIVSYEKP KPVIGFHRYV FLLFKQRDRF

            ....|....| ....|....| ....|..
                 105        115        125
TFL1_NEU    NTR~FAEEND LGLPVAAVYF NAQRETA
TFL1_REP    NTRKFAEEND LGLPVAAVYF NAQRETA
AaFTL2      NTRAFAQEND LGLPVAAIYF NAQRENA
```

**Figure S6. Alignment of functional consensus sequences and *A. artemisiifolia* FTL2.** TFL1-clade repressor consensus (TFL1_REP), TFL1-clade neutral consensus (TFL1_NEU), and *Aa*FTL2. Consensus sequences are based on 33 and 6 sequences, respectively. Yellow highlights for residues unique to the neutral consensus, red for residues unique to the repressor consensus, grey for residues not matching any consensus, and in purple the histidine residue that confers floral repressive function to *Arabidopsis thaliana* TFL1. Tildes indicate positions without a predominant residue.

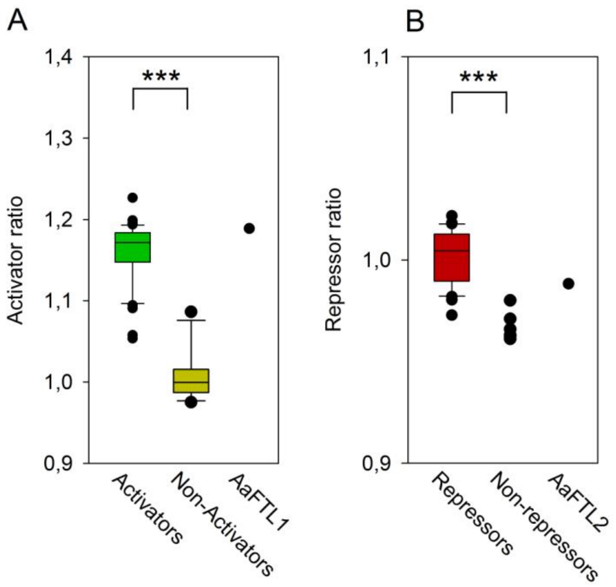


**Figure S7. Activator and repressor ratios.** (A) Activator ratio of proteins within the FT-clade based on 132 informative amino acid positions; 44 activator sequences and 12 non-activator sequences were used. (B) Repressor ratio, based on 132 informative positions; 33 repressor sequences and 6 non-repressor sequences were used. Box plots show the first, second and third quartiles (the box), the 10th and 90th percentiles (the whiskers), and the outliers as individual dots. Mann-Whitney U tests were performed to test for significant differences between groups. Significant differences are denoted with asterisks (ns p>0.05, * p<0.05, ** p<0.01, *** p<0.001).

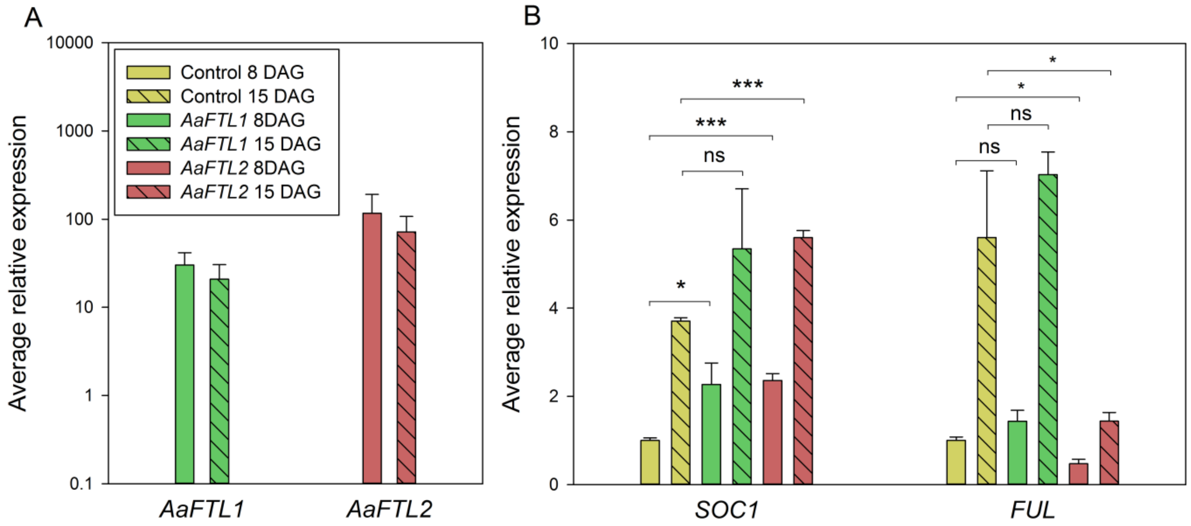


**Figure S8. Gene expression in transgenic Arabidopsis plants expressing *AaFTL1* and *AaFTL2*.** Average relative expression of transgenes (A) and endogenous genes (B) in *AaFTL1* and *AaFTL2* expressing lines and the related control at 8 DAG and 15 DAG. The results presented here are averages of three independent insertion lines for each transformation. For each time point 10-12 seedlings were pooled per line.

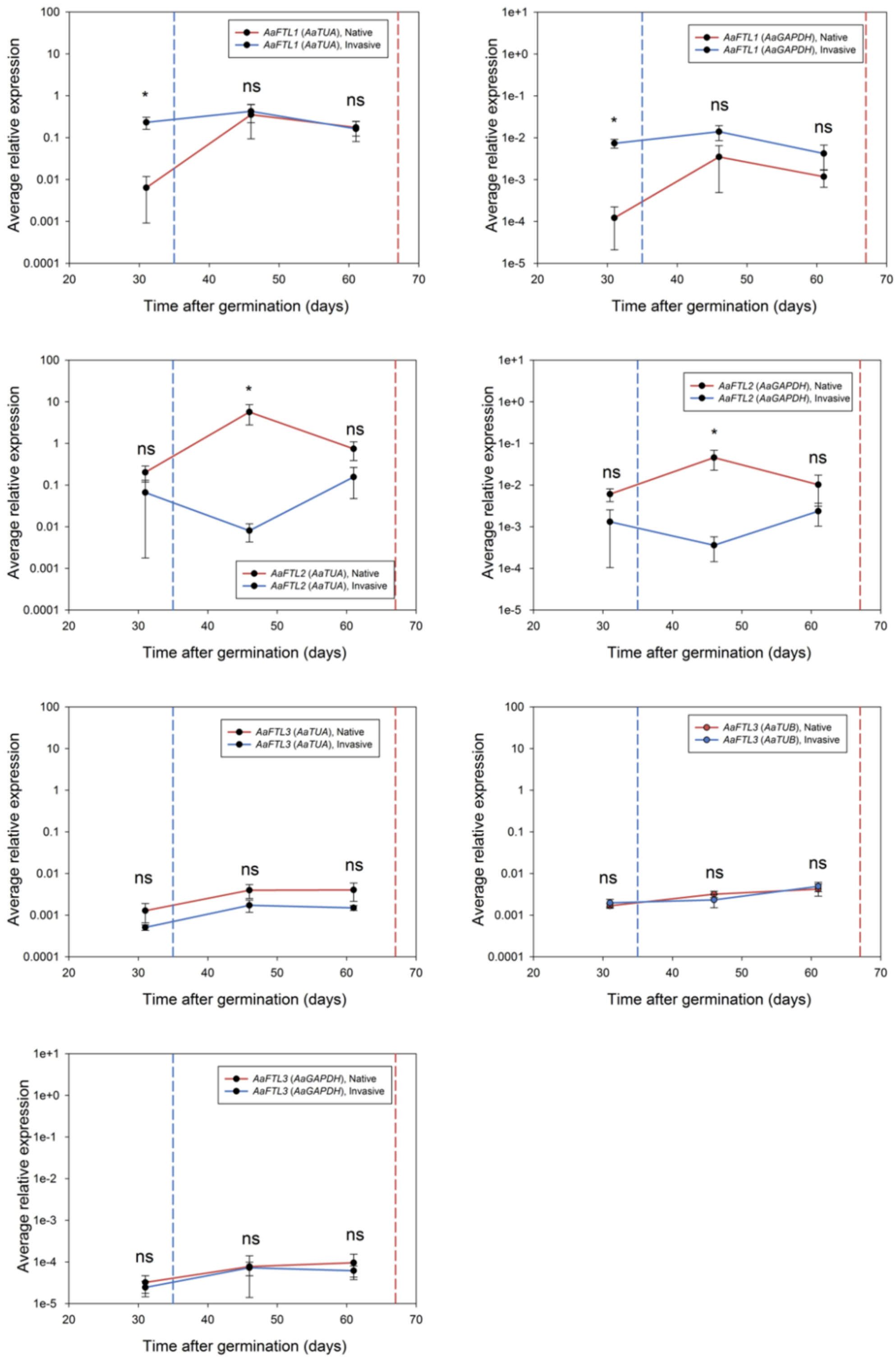


**Figure S9. Average relative expression of *A. artemisiifolia AaFTL1*, *AaFTL2*, and *AaFTL3*.** Data points indicate average expression values of invasive plants (n=5; blue) and native plants (n=4; red). Error bars show the SEM. Between brackets the used reference gene is indicated. Mann-Whitney U tests were performed to check for significant differences between groups; significant differences (between native and invasive) are denoted with asterisks (ns $p>0.05$, * $p<0.05$). The dashed lines indicate the average time when flowering was initiated for the invasive population (blue) and the native population (red).

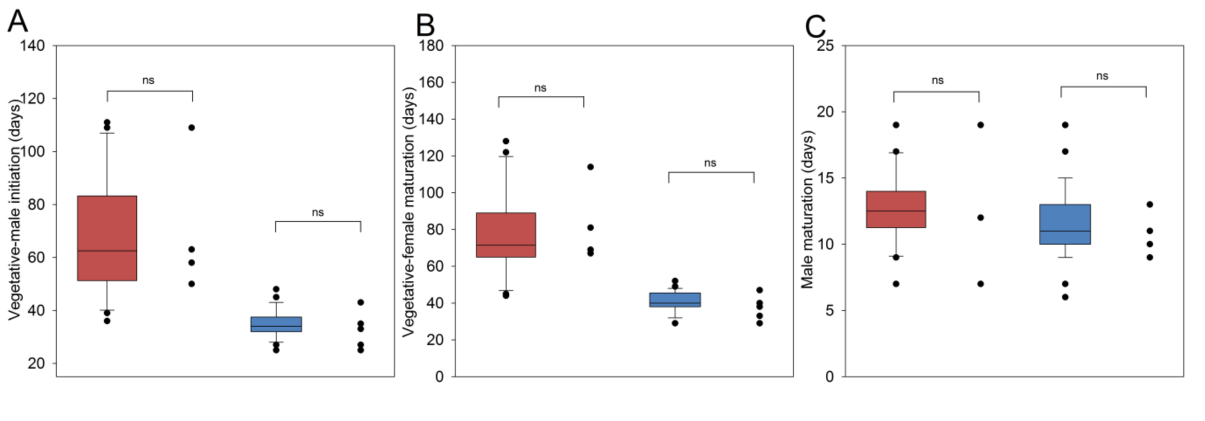


**Figure S10. Comparison of flowering phenology of total and sample populations.** The box plots here are made with the data of all plants of the respective population (red: native, blue: invasive), and the scatter plots right next to the box plots are made with only those individuals that have been used for expression analysis. Box plots show the first, second and third quartiles (the box), the 10th and 90th percentiles (the whiskers), and the outliers as individual dots. Mann-Whitney U tests were performed to check for significant differences between groups (ns $p>0.05$): invasive vegetative-male initiation $p=0.47$, native vegetative-male initiation $p=1.00$, invasive male maturation $p=0.41$, native male maturation $p=0.85$, invasive vegetative-female maturation $p=0.37$, native vegetative-female maturation $p=0.61$.

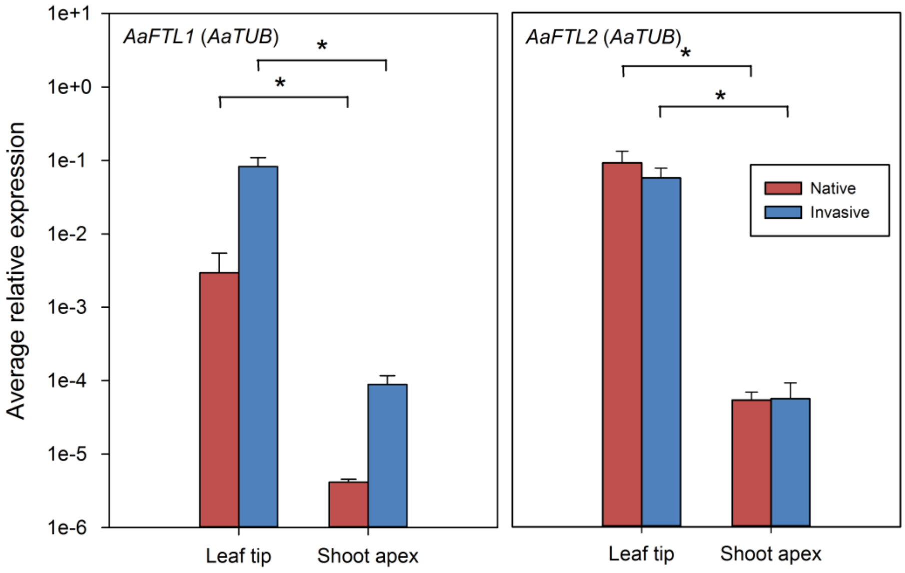


**Figure S11. *AaFTL1* and *AaFTL2* expression in leaves and shoot apices.** Expression of *AaFTL1* (left) and *AaFTL2* (right) in leaf tips and shoot apices of native (red), and invasive (blue) plants. Each bar represents the average of four pools of four plants each. Mann-Whitney U tests were performed to test for significant differences between groups. Significant differences are denoted with asterisks (* $p<0.05$).

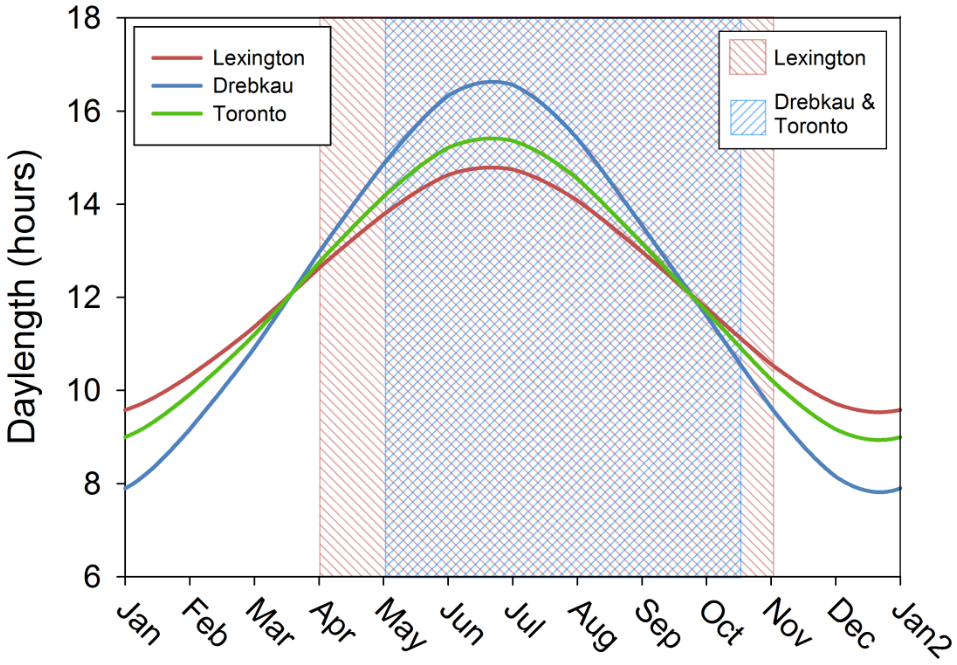


**Figure S12. Day length and vegetation period at the collection places of the two studied *A. artemisiifolia* populations.** The blue hatching indicates the length of the vegetation period (i.e. the time with an average minimum temperature above 5°C) for Holzdorf (Sachsen-Anhalt, Germany), near the collection place of the invasive population in Drebkau, and for Toronto (Ontario, Canada) near the Northern border of the native range. The red hatching indicates the length of the vegetation period for Blue grass airport (Kentucky, USA) near the collection place of the native population in Lexington. The solid lines indicate the day length in Lexington (red), Drebkau (blue), and Toronto (green) (http://timeanddate.com, access date: 10 February 2017).

## Supplementary Tables

### Table S1. Primers used in this study.

| Sequence (5' -> 3') | Function | Reference |
|---|---|---|
| TGCAGAGGGCTGTTTGCATGA | *AaTUA* qPCR forward | (El Kelish *et al.*, 2014) |
| ACCCACATACCAGTGAACAAAAG | *AaTUA* qPCR reverse | (El Kelish *et al.*, 2014), 7G>A |
| ATCGACGTAACCGGCAAGTACA | *AaTUB* qPCR forward | (Li *et al.*, 2015) |
| AGGCTCCAGATCCATGAGGAC | *AaTUB* qPCR reverse | (Li *et al.*, 2015) |
| ATTGACCTCCTCAGCGAAAG | *AaGAPDH* qPCR forward | (Hodgins *et al.*, 2013) |
| TGTGTTGCCCTCACTCAAAG | *AaGAPDH* qPCR reverse | (Hodgins *et al.*, 2013) |
| GACTGATATTCCAGCTACCACAGGT | *AaFTL1* qPCR forward, *AaFTL1* 3' RACE, OX genotyping forward | (Li *et al.*, 2015) |
| GTCGAAACAACACGAAAACCATAC | *AaFTL1* qPCR reverse, *AaFTL1* 5' RACE, OX genotyping reverse | (Li *et al.*, 2015) |
| GAATGTCTGTGACAATCCAATGA | *AaFTL2* qPCR forward, OX genotyping forward | |
| TTGCCTCTAAACCTCGTGTTC | *AaFTL2* qPCR reverse, OX genotyping reverse | |
| GCCTAATCTCATTACTTCTAAACCTCA | *AaFTL3* qPCR forward | |
| GGGTATAGAGACAGAGAATTACAATGG | *AaFTL3* qPCR reverse | |
| TGGCCTTGGACTCTCATAACACACGA | *AaFTL1* 5' RACE nested | |
| TCCAGCTACCACAGGTGCACGATTT | *AaFTL1* 3' RACE nested | |
| ATCTCGTGAAACCGGTGGCCTCACT | *AaFTL2* 5' RACE | |
| TGGCCGTTAGCAACCGGGCAATGG | *AaFTL2* 5' RACE nested | |
| GTTGTAGGCGGGTGATAGGCGATGT | *AaFTL2* 3' RACE | |
| GACCAATTCACACCGAGTGCGCAAA | *AaFTL2* 3' RACE nested | |
| GGGGACAAGTTTGTACAAAAAAGCAGGCT TAATGTCCAGGGGAAGGGATCCA | *AaFTL1* cloning forward | |
| GGGGACCACTTTGTACAAGAAAGCTGGGT CTTATCTCCTTCGTCCACCAAAC | *AaFTL1* cloning reverse | |
| GGGGACAAGTTTGTACAAAAAAGCAGGCT TCAGCATGTCACTTGTTGTAGG | *AaFTL2* cloning forward | |
| GGGGACCACTTTGTACAAGAAAGCTGGGT ATTATCTTCTGCGAGGGGCATT | *AaFTL2* cloning reverse | |
| TAACGTGGCCAAAATGATGC | *PP2AA3* qPCR forward | |
| GTTCTCCACAACCGCTTGGT | *PP2AA3* qPCR reverse | |
| TCACAACAATTCGCTTCTCAA | *FUL* qPCR forward | |
| TTGGACTAATTGTCCTTCTTGCT | *FUL* qPCR reverse | |
| AGCAGCTCAAGCAAAAGGAG | *SOC1* qPCR forward | |
| TTGACCAAACTTCGCTTTCA | *SOC1* qPCR reverse | |

## Table S2. *A. artemisiifolia FT/TFL1* cDNA sequences

| Name | Genbank accession | Sequence |
|---|---|---|
| *AaFTL1* | KY548398 | GAAAAAACCATTAGACGAACTACATACTTGAAAGAATAAACTCGATTTTTCATAGATTGAGAAAATAAGTCCTTTTTTTATATAATGTCCAGGGGAAGG<br>GATCCATTAGTCGTTGGACGCGTGATAGGAGATGTTCTTGAGAGTTTCACCAAGTCGATGAACCTTACTGTGTCGTATAACGATAGGGAAGTTAGCAAT<br>GGATGCGAGCTAAAACCCTcTCAAGTTGTGAACCAACCAAGGGTTGAAATTGGAGGCGACGACTTGCGCGCTTTTCACACTCTAGTAATGGTGGATCCTG<br>ATGCTCCAAGTCCAAGTGACCCTAACCTTAGGGAATACTTGCATTGGTTGGTGACTGATATTCCAGCTACCACAGGTGCaCGwTTTGGTCAAGAAGTsGT<br>GTGtTAtGAGAGTCCAAGgCCAtCgATGGGAATTCATCGTATGGTTTTCGTSTTGTTTCGACAGTTGGGTCGACAAACTGTGTATGCCCCAGGGTGGCGCC<br>AGAACTTCAACACAAAAGACTTTGCTGAGCTCTATAACCTTGGATCGCCTGTGGCTGCGGTCTACTTCAATTGCCAACGCGAAAGTGGGTTTGGTGGACG<br>AAGGAGATAAGAATAAGATGAAAGCATGTCAATGGTATGCAAATGACGTCATAAATGCACGTTTAAAGCGTGTACAGCATTTTATTATAGCCTTATTTG<br>TATTAAGTTATATATTATTATACATATACATACATATAAAGAAGAGGGGGTGACAAGGACGAATAGCTATAAAGGAATAAGAATACTTTCTGTGTAAT<br>GCTTAGTAAGAATAGAAATGATACAAAAATGATTATATGAAATAACATATATCTCTGTTAGCCGAAAAAAAAAAAAAAAAAAAAAAA |
| *AaFTL2* | KY548399 | GAAaATCCACAAAAYAAaTATAAGAGTATATCAGCATGTCACTTGTTGTAGGGCGGGTGATAGGCGATGTCGTCGACCAATTCACACCGAGTGCGCAAAT<br>GGATGTAATCTATAATCCCCATTGCCCGGTtGCTAACGGCCATGAGCTCAAGCCTAATCTCGTTGcCTCTAAACCTCGTGTTCATATCGGCGGTGTTGACA<br>TGAGATCtTCTTATACTCTAGTcATGACtGATCCGGATGCTCCAAGTCCAAGTGATCCGTACTTGAGAGAACATCTTCATTGGATTGTCACAGACATTCCG<br>GGTACAACTGATGCAACTTTCGGAAGGGAGATTGTGAGCTATGAAAAACCAAAGCCAGTGATCGGATTCCATAGGTATGTGTTCTTATTGTTCAAGCAA<br>AGAGCTAGGCAATCAGTGAGGCCACCGGTTTCACGAGATCGATTCAACACTCGTGCCTTCGCTcAAGAAAATGACTTGGGGTTACCTGTTGCTGCTATCT<br>ACTTCAATGCTCAAAGAGAaAATGCCCCTCGCAGAAGATAATTCAATTTCATGATaGATATACTAAGAAATAATAAGAAATAGTATAAGGCTAAATAAA<br>ACATGTTAGTTGTGGCTTCCATTATGCATTTTACTATCTTCCTGAGATATGTATTTTGCTAATTCCTTGTTTTAAGTTTCTTTGTAATGATTTGAACTAC<br>TTTTAAAGTTCAAGTAATCAAATAAATATATTATTTATTGGATGTTTTCCAATTTAAAAAaAAAAAAAAAAAAAAAAAA |
| *AaFTL3** | | GAGCTGATGCCTAATCTCATTACTTCTAAACCTCAGGTTCATGTTGGTGGTGTTGACATGAGAACTTCTTATACCATTGTAATTCTCTGTCTCTATACCC<br>CCCCACCCCCAAAAAAAAaTCTACATTTATTAACGTACTAACCATGTTTCCATTTTTTACCTGAAGATCTTGACCGACCCGGATGCACCGAGTCCAAGTG<br>ATCCTCACTTGAGAGAACATCTCCATTGGTGAAGACATGTTTAAAAGACCGTTCTATTGGTCAAAAGAACTATTTCTTAGAATAGTTGGTGATATGGAG<br>TGAAACTTTGAATGGTTTGAAACAAGTTGGGATGCCATATACGATAAGAATTTTACATCACTACAGAAGTGCACAAAGGTTATCTGTCAACTTGTAGAC<br>CAAGAACACATGAATAAAATGCGGTTTACTCTAAAAAAAAAAAAAAAAAAAAAAa |

* Sequence is incomplete; 5' end is missing.

## Table S3. List of FT-like proteins with known and predicted function.

| Protein name | Reported function | Activator ratio* Predicted function | Reference** |
|---|---|---|---|
| >BAH24199.1_FT-like_protein_3_Hordeum_vulgare_subsp._vulgare | Activator | 1.057595 | (Kikuchi *et al.*, 2009) |
| >ABW96231.1_ZCN8_Zea_mays | Activator | 1.053482 | (Meng *et al.*, 2011) |
| >AJF40163.1_FT5a_Glycine_max | Activator | 1.090635 | (Kong *et al.*, 2010) |
| >AEI99555.1_FTc_Medicago_truncatula | Activator | 1.099094 | (Laurie *et al.*, 2011) |
| >AGZ20208.1_FT2_Allium_cepa | Activator | 1.121969 | (Lee *et al.*, 2013) |
| >AEI99553.1_FTb1_Medicago_truncatula | Activator | 1.094273 | (Laurie *et al.*, 2011) |
| >BAA77840.1_TSF_Arabidopsis_thaliana | Activator | 1.116733 | (Yamaguchi *et al.*, 2005) |
| >OAP18553.1_RSB8_Arabidopsis_thaliana | Activator | 1.116346 | (Kardailsky *et al.*, 1999) |
| >AGZ20207.1_FT1_Allium_cepa | Activator | 1.144917 | (Lee *et al.*, 2013) |
| >AEI99551.1_FTa1_Medicago_truncatula | Activator | 1.143048 | (Laurie *et al.*, 2011) |
| >AFS17372.1_flowering_locus_T4_Nicotiana_tabacum | Activator | 1.107591 | (Harig *et al.*, 2012) |
| >BAV67095.1_PEBP_protein_Solanum_tuberosum | Activator | 1.155353 | (Navarro *et al.*, 2011) |
| >OsFTL1_(LOC_Os01g11940) | Activator | 1.161129 | (Izawa *et al.*, 2002) |
| >ABB99414.1_FT-like_protein_Hordeum_vulgare_subsp._vulgare | Activator | 1.154941 | (Kikuchi *et al.*, 2009) |
| >AJF40158.1_FT2a_Glycine_max | Activator | 1.173936 | (Kong *et al.*, 2010) |
| >AAZ38709.1_FT-like_protein_Hordeum_vulgare_subsp._vulgare | Activator | 1.198744 | (Kikuchi *et al.*, 2009) |
| >ABK32208.1_VRN3_Triticum_aestivum | Activator | 1.195302 | (Yan *et al.*, 2006) |
| >BAB78480.1_FT-like_protein_Oryza_sativa_Japonica_Group | Activator | 1.177019 | (Komiya *et al.*, 2008) |
| >BAB61028.1_Hd3a_Oryza_sativa_Japonica_Group | Activator | 1.18669 | (Kojima *et al.*, 2002; Komiya *et al.*, 2008) |
| >BAB61029.1_Hd3a_Oryza_sativa_Indica_Group | Activator | 1.18669 | (Kojima *et al.*, 2002) |
| >ADI58462.1_flowering_locus_T_Cymbidium_goeringii | Activator | 1.163065 | (Xiang *et al.*, 2012) |
| >AGK89939.1_flowering_locus_T_Actinidia_chinensis | Activator | 1.168687 | (Varkonyi-Gasic *et al.*, 2013) |
| >ADM92610.1_flowering_locus_T-like_protein_FT2_Beta_vulgaris | Activator | 1.16449 | (Pin *et al.*, 2010) |
| >ABI94605.1_flowering_locus_T-like_1_Cucurbita_maxima | Activator | 1.170347 | (Lin *et al.*, 2007) |
| >ABI94606.1_flowering_locus_T-like_2_Cucurbita_maxima | Activator | 1.181649 | (Lin *et al.*, 2007) |
| >AAO31792.1_SP3D_Solanum_lycopersicum | Activator | 1.167807 | (Lifschitz *et al.*, 2006) |
| >BAV67096.1_PEBP_protein_Solanum_tuberosum | Activator | 1.167984 | (Navarro *et al.*, 2011) |
| >ADF32946.1_flowering_locus_T1_Helianthus_annuus | Activator | 1.182241 | (Blackman *et al.*, 2010) |
| >ADF32947.1_flowering_locus_T2_Helianthus_annuus | Activator | 1.184487 | (Blackman *et al.*, 2010) |
| >ACY82397.2_FTL_Chrysanthemum_lavandulifolium | Activator | 1.18511 | (Fu *et al.*, 2014) |
| >BAL14659.1_FT_like_protein_Chrysanthemum_seticuspe_f._boreale | Activator | 1.174438 | (Oda *et al.*, 2012) |
| >ADF32945.1_flowering_locus_T4_Helianthus_annuus | Activator | 1.192548 | (Blackman *et al.*, 2010) |
| >ABI99465.1_FT-like_protein_Vitis_vinifera | Activator | 1.160259 | (Carmona *et al.*, 2007) |
| >BAG12903.1_hypothetical_protein_Populus_nigra | Activator | 1.226506 | (Igasaki *et al.*, 2008) |
| >AEP23098.1_flowering_locus_T1_Fragaria_vesca | Activator | 1.173706 | (Koskela *et al.*, 2012) |
| >BAD08340.1_flowering_locus_T_like_protein_Malus_domestica | Activator | 1.186782 | (Kotoda *et al.*, 2010) |
| >BAI77728.1_flowering_locus_T_like_protein_Malus_domestica | Activator | 1.180417 | (Kotoda *et al.*, 2010) |
| >BAD02371.1_flowering_locus_T_Populus_nigra | Activator | 1.173275 | (Igasaki *et al.*, 2008) |
| >XP_002311264.1_FTL_protein_Populus_trichocarpa | Activator | 1.172826 | (Böhlenius *et al.*, 2006) |
| >BAD01561.1_flowering_locus_T_Populus_nigra | Activator | 1.176259 | (Igasaki *et al.*, 2008) |
| >BAD01612.1_flowering_locus_T_Populus_nigra | Activator | 1.1768 | (Igasaki *et al.*, 2008) |
| >JcFT | Activator | 1.168796 | (Ye *et al.*, 2014) |
| >ARE72515.1_FT2_Actinidia_chinensis_var._chinensis | Activator | 1.193687 | (Voogd *et al.*, 2017) |
| >ARE72514.1_FT1_Actinidia_chinensis_var._chinensis | Activator | 1.186753 | (Voogd *et al.*, 2017) |
| >ABW96244.1_ZCN26_Zea_mays | Neutral | 1.003996 | (Meng *et al.*, 2011) |
| >ABW96230.1_ZCN7_Zea_mays | Neutral | 1.051784 | (Meng *et al.*, 2011) |
| >AGZ20212.1_FT6_Allium_cepa | Neutral | 0.995625 | (Lee *et al.*, 2013) |
| >ABW96240.1_ZCN18_Zea_mays | Neutral | 0.975101 | (Meng *et al.*, 2011) |
| >AGZ20209.1_FT3_Allium_cepa | Neutral | 0.992696 | (Lee *et al.*, 2013) |
| >AGZ20211.1_FT5_Allium_cepa | Neutral | 0.980947 | (Lee *et al.*, 2013) |
| >AGZ20210.1_FT4_Allium_cepa | Repressor | 0.99284 | (Lee *et al.*, 2013) |
| >AAO31793.1_SP5G_Solanum_lycopersicum | Repressor | 1.01732 | (Cao *et al.*, 2016) |
| >AFS17370.1_flowering_locus_T2_Nicotiana_tabacum | Repressor | 0.985027 | (Harig *et al.*, 2012) |
| >AFS17369.1_flowering_locus_T1_Nicotiana_tabacum | Repressor | 1.010092 | (Harig *et al.*, 2012) |
| >AFS17371.1_flowering_locus_T3_Nicotiana_tabacum | Repressor | 1.003454 | (Harig *et al.*, 2012) |
| >ADM92608.1_flowering_locus_T-like_protein_FT1_Beta_vulgaris | Repressor | 1.086107 | (Pin *et al.*, 2010) |
| >OsFTL8_(LOC_Os01g10590) | Unknown | 1.056524 | |
| >OsFTL13_(LOC_Os02g13830) | Unknown | 1.066372 | |
| >OsFTL12_(LOC_Os06g35940) | Unknown | 0.993287 | |
| >OsFTL10_(LOC_Os05g44180) | Unknown | 1.032997 | |
| >OsFTL9_(LOC_Os01g54490) | Unknown | 1.077175 | |

| | | | |
|---|---|---|---|
| >OsFTL4_(LOC_Os09g33850) | Unknown | 0.984624 | |
| >NP_001296779.1_protein_TWIN_SISTER_of_FT_Zea_mays | Unknown | 1.053934 | |
| >OsFTL7_(LOC_Os12g13030) | Unknown | 1.059814 | |
| >ABW96238.1_ZCN16_Zea_mays | Unknown | 1.052848 | |
| >OsFTL5_(LOC_Os02g39064) | Unknown | 1.055192 | |
| >OsFTL6_(LOC_Os04g41130) | Unknown | 1.050723 | |
| >ABW96241.1_ZCN19_Zea_mays | Unknown | 1.031632 | |
| >ABW96243.1_ZCN25_Zea_mays | Unknown | 1.041056 | |
| >ABW96239.1_ZCN17_Zea_mays | Unknown | 1.047656 | |
| >OsFTL11_(LOC_Os11g18870) | Unknown | 1.07817 | |
| >AJF40164.1_FT5b_Glycine_max | Unknown | 1.081159 | |
| >AJF40156.1_FT1a_Glycine_max | Unknown | 1.062158 | |
| >AJF40157.1_FT1b_Glycine_max | Unknown | 1.107233 | |
| >AJF40165.1_FT6_Glycine_max | Unknown | 1.130352 | |
| >AEI99554.1_FTb2_Medicago_truncatula | Unknown | 1.10714 | |
| >AJF40162.1_FT4_Glycine_max | Unknown | 1.10119 | |
| >AEI99552.1_FTa2_Medicago_truncatula | Unknown | 1.118537 | |
| >AJF40160.1_FT3a_Glycine_max | Unknown | 1.129797 | |
| >AJF40161.1_FT3b_Glycine_max | Unknown | 1.143779 | |
| >ABW96236.1_ZCN14_Zea_mays | Unknown | 1.159748 | |
| >AJF40159.1_FT2b_Glycine_max | Unknown | 1.16524 | |
| >ABW96237.1_ZCN15_Zea_mays | Unknown | 1.186119 | |
| >ABR20498.1_FTL1_Cucurbita_moschata | Unknown | 1.17163 | |
| >ABR20499.1_FTL2_Cucurbita_moschata | Unknown | 1.183432 | |
| >**AaFTL1** | Unknown | 1.188732 | |
| >BAL14657.1_FT_like_protein_Chrysanthemum_seticuspe_f._boreale | Unknown | 1.171199 | |
| >BAL14658.1_FT_like_protein_Chrysanthemum_seticuspe_f._boreale | Unknown | 1.175306 | |
| >AEO72030.1_flowering_locus_T_protein_Prunus_persica | Unknown | 1.188798 | |
| >BAG12904.1_FLOWERING_LOCUS_T_Populus_nigra | Unknown | 1.173275 | |
| >XP_002316173.1_FTL_protein_Populus_trichocarpa | Unknown | 1.176259 | |
| >ABW96235.1_ZCN12_Zea_mays | Unknown | 1.073423 | |

*An activator ratio of 1.083 was set as the cut-off value for activator activity: equal or higher values indicate predicted activator function (green shading), and lower values indicate non-activator function (yellow shading).
**References to functional analyses of the proteins.

## Table S4. List of TFL1-like proteins with known and predicted function

| Protein name | Reported function | Repressor ratio* Predicted function | Reference** |
|---|---|---|---|
| >OAO92261.1_BFT_Arabidopsis_thaliana | Repressor | 0.9948 | (Yoo *et al.*, 2010) |
| >AAQ20811.1_late-flowering_Pisum_sativum | Repressor | 1.0140 | (Foucher *et al.*, 2003) |
| >ARE72517.1_CEN2_Actinidia_chinensis_var._chinensis | Repressor | 1.0127 | (Voogd *et al.*, 2017) |
| >ARE72518.1_CEN3_Actinidia_chinensis_var._chinensis | Repressor | 1.0099 | (Voogd *et al.*, 2017) |
| >OAO93664.1_TFL1_Arabidopsis_thaliana | Repressor | 1.0067 | (Bradley *et al.*, 1997) |
| >ACL27223.1_fasciculate_Capsicum_frutescens | Repressor | 0.9803 | (Elitzur *et al.*, 2009) |
| >AAD42896.1_Cen-like_protein_FDR1_Oryza_sativa_Indica_Group | Repressor | 0.9829 | (Zhang *et al.*, 2005) |
| >AAD42895.1_Cen-like_protein_FDR2_Oryza_sativa_Indica_Group | Repressor | 0.9831 | (Nakagawa *et al.*, 2002; Zhang *et al.*, 2005) |
| >ABW96224.1_ZCN1_Zea_mays | Repressor | 0.9728 | (Danilevskaya *et al.*, 2010) |
| >ABW96227.1_ZCN4_Zea_mays | Repressor | 1.0050 | (Danilevskaya *et al.*, 2010) |
| >ABW96228.1_ZCN5_Zea_mays | Repressor | 1.0044 | (Danilevskaya *et al.*, 2010) |
| >ABW96225.1_ZCN2_Zea_mays | Repressor | 0.9886 | (Danilevskaya *et al.*, 2010) |
| >RCN2_(LOC_Os02g32950) | Repressor | 0.9971 | (Nakagawa *et al.*, 2002) |
| >BAI66119.1_terminal_flower_1b_Glycine_max | Repressor | 0.9918 | (Liu *et al.*, 2010) |
| >AFI47669.1_TFLly_Phaseolus_vulgaris | Repressor | 0.9916 | (Repinski *et al.*, 2012) |
| >ARE72516.1_CEN1_Actinidia_chinensis_var._chinensis | Repressor | 1.0182 | (Voogd *et al.*, 2017) |
| >ADO64258.1_TFL1-like_protein_Fragaria_vesca | Repressor | 1.0132 | (Koskela *et al.*, 2012) |
| >ADL62867.1_terminal_flower_1_Prunus_persica | Repressor | 1.0045 | (Chen *et al.*, 2013) |
| >BAD06418.1_TFL1-like_protein_Malus_domestica | Repressor | 1.0146 | (Kotoda *et al.*, 2006; Mimida *et al.*, 2009) |
| >BAG31959.1_TFL1_like_protein_Malus_domestica | Repressor | 1.0111 | (Mimida *et al.*, 2009) |
| >BAN89466.1_TFL1_family_protein_Chrysanthemum_seticuspe_f._boreale | Repressor | 0.9952 | (Higuchi and Hisamatsu, 2015) |
| >BAA75933.1_ATC_Arabidopsis_thaliana | Repressor | 0.9906 | (Huang *et al.*, 2012) |
| >AGK89940.1_centroradialis_Actinidia_deliciosa | Repressor | 1.0128 | (Varkonyi-Gasic *et al.*, 2013) |
| >ARE72519.1_CEN4_Actinidia_chinensis_var._chinensis | Repressor | 1.0074 | (Voogd *et al.*, 2017) |
| >ABI99466.1_TFL1A_protein_Vitis_vinifera | Repressor | 1.0098 | (Carmona *et al.*, 2007) |
| >BAG31957.1_CENTRORADIALIS_like_protein_Malus_domestica | Repressor | 1.0112 | (Mimida *et al.*, 2009) |
| >BAD22601.1_flowering_locus_T_like_protein_Populus_nigra | Repressor | 1.0216 | (Igasaki *et al.*, 2008) |
| >BAD22599.1_terminal_flower_1_Populus_nigra | Repressor | 1.0175 | (Igasaki *et al.*, 2008) |
| >XP_006384827.1_TFL1a_family_protein_Populus_trichocarpa | Repressor | 1.0178 | (Mohamed *et al.*, 2010) |
| >BAN89465.1_AFT_Chrysanthemum_seticuspe_f._b | Repressor | 0.9967 | (Higuchi *et al.*, 2013) |
| >ARE72520.1_BFT1_Actinidia_chinensis_var._chinensis | Repressor | 0.9818 | (Voogd *et al.*, 2017) |
| >ARE72522.1_BFT3_Actinidia_chinensis_var._chinensis | Repressor | 0.9865 | (Voogd *et al.*, 2017) |
| >ARE72521.1_BFT2_Actinidia_chinensis_var._chinensis | Repressor | 0.9862 | (Voogd *et al.*, 2017) |
| >AAC26161.1_self-pruning_protein_Solanum_lycopersicum | Neutral | 0.9709 | (Pnueli *et al.*, 1998) |
| >ABW96229.1_ZCN6_Zea_mays | Neutral | 0.9611 | (Danilevskaya *et al.*, 2010) |
| >ABW96226.1_ZCN3_Zea_mays | Neutral | 0.9658 | (Danilevskaya *et al.*, 2010) |
| >AAR03725.1_TFL1a_Pisum_sativum | Neutral | 0.9800 | (Foucher *et al.*, 2003) |
| >CAC21564.1_centroradialis_Antirrhinum_majus | Neutral | 0.9630 | (Bradley *et al.*, 1996; Cremer *et al.*, 2001) |
| >BAD08339.1_flowering_locus_T_like_protein_Populus_nigra | Neutral | 0.9625 | (Igasaki *et al.*, 2008) |
| >ADM92612.1_centroradialis-like_protein_CEN1_Beta_vulgaris | Unknown | 0.9857 | |
| >AAO31795.1_SP9D_Solanum_lycopersicum | Unknown | 1.0116 | |
| >BAH24197.1_homologous_protein_to_TFL1_Hordeum_vulgare_subsp._vulgare | Unknown | 0.9739 | |
| >RCN4_(LOC_Os04g33570) | Unknown | 1.0006 | |
| >BAI66120.1_terminal_flower_1a_Glycine_max | Unknown | 0.9912 | |
| >ABI99467.1_TFL1B_protein_Vitis_vinifera | Unknown | 1.0093 | |
| >ADO61015.1_terminal_flower_1_Helianthus_annuus | Unknown | 0.9855 | |
| >BAG31958.1_CENTRORADIALIS_like_protein_Malus_domestica | Unknown | 1.0083 | |
| >XP_002312811.1_TFL1a_family_protein_Populus_trichocarpa | Unknown | 1.0214 | |
| >**AaFTL2** | Unknown | 0.9883 | |
| >HaBFT_(DY918510) | Unknown | 0.9822 | |
| >ADM92614.1_brother_of_FT_AND_TFL1-like_protein_BFT1_Beta_vulgaris | Unknown | 0.9684 | |
| >ABI99468.1_TFL1C_protein_Vitis_vinifera | Unknown | 0.9896 | |
| >MdBFT_(EB138045) | Unknown | 0.9639 | |

*A repressor ratio of 0.9801 was used as a cut-off for repressor activity: equal or higher indicates repressor function (red shading), lower indicates non-repressor function (yellow shading).
**References to functional analyses of the proteins.

**Table S5. Standard errors of gene expression values for different reference genes as established by RT-qPCR.**

| | ***AaTUA*** | ***AaTUB*** | ***AaGAPDH*** |
|---|---|---|---|
| **$SEM_1$*** | 4.28E-08 | 2.10E-08 | 2.02E-06 |
| **$SEM_2$*** | 4.18E-08 | 2.38E-08 | 1.98E-06 |

* The SEMs were obtained by first calculating expression values (=$E^{-Ct}$) using predetermined primer efficiencies (E) for the gene in all plants (from both native and invasive populations) and time points. Variances across expression values were then determined for each time point to calculate $SEM_1$ or for each plant to calculate $SEM_2$.

## Supplementary References

**Blackman BK, Strasburg JL, Raduski AR, Michaels SD, Rieseberg LH**. 2010. The role of recently derived FT paralogs in sunflower domestication. *Current Biology* **20**, 629-635.

**Böhlenius H, Huang T, Charbonnel-Campaa L, Brunner AM, Jansson S, Strauss SH, Nilsson O**. 2006. CO/FT regulatory module controls timing of flowering and seasonal growth cessation in trees. *science* **312**, 1040-1043.

**Bradley D, Carpenter R, Copsey L, Vincent C**. 1996. Control of inflorescence architecture in Antirrhinum. *Nature* **379**, 791.

**Bradley D, Ratcliffe O, Vincent C, Carpenter R, Coen E**. 1997. Inflorescence commitment and architecture in Arabidopsis. *science* **275**, 80-83.

**Cao K, Cui L, Zhou X, Ye L, Zou Z, Deng S**. 2016. Four tomato FLOWERING LOCUS T-like proteins act antagonistically to regulate floral initiation. *Frontiers in plant science* **6**, 1213.

**Carmona MJ, Calonje M, Martinez-Zapater JM**. 2007. The FT/TFL1 gene family in grapevine. *Plant Molecular Biology* **63**, 637-650.

**Chen Y, Jiang P, Thammannagowda S, Liang H, Wilde HD**. 2013. Characterization of peach TFL1 and comparison with FT/TFL1 gene families of the Rosaceae. *Journal of the American Society for Horticultural Science* **138**, 12-17.

**Cremer F, Lönnig W-E, Saedler H, Huijser P**. 2001. The delayed terminal flower phenotype is caused by a conditional mutation in the CENTRORADIALIS gene of snapdragon. *Plant Physiology* **126**, 1031-1041.

**Danilevskaya ON, Meng X, Ananiev EV**. 2010. Concerted modification of flowering time and inflorescence architecture by ectopic expression of TFL1-like genes in maize. *Plant Physiology* **153**, 238-251.

**El Kelish A, Zhao F, Heller W, Durner J, Winkler JB, Behrendt H, Traidl-Hoffmann C, Horres R, Pfeifer M, Frank U**. 2014. Ragweed (Ambrosia artemisiifolia) pollen allergenicity: SuperSAGE transcriptomic analysis upon elevated CO 2 and drought stress. *BMC Plant Biology* **14**, 176.

**Elitzur T, Nahum H, Borovsky Y, Pekker I, Eshed Y, Paran I**. 2009. Co-ordinated regulation of flowering time, plant architecture and growth by FASCICULATE: the pepper orthologue of SELF PRUNING. *Journal of Experimental Botany* **60**, 869-880.

**Foucher F, Morin J, Courtiade J, Cadioux S, Ellis N, Banfield MJ, Rameau C**. 2003. DETERMINATE and LATE FLOWERING are two TERMINAL FLOWER1/CENTRORADIALIS homologs that control two distinct phases of flowering initiation and development in pea. *Plant Cell* **15**, 2742-2754.

**Fu J, Wang L, Wang Y, Yang L, Yang Y, Dai S**. 2014. Photoperiodic control of FT-like gene ClFT initiates flowering in Chrysanthemum lavandulifolium. *Plant Physiology and Biochemistry* **74**, 230-238.

**Harig L, Beinecke FA, Oltmanns J, Muth J, Muller O, Ruping B, Twyman RM, Fischer R, Prufer D, Noll GA**. 2012. Proteins from the FLOWERING LOCUS T-like subclade of the PEBP family act antagonistically to regulate floral initiation in tobacco. *The Plant Journal* **72**, 908-921.

**Higuchi Y, Hisamatsu T**. 2015. CsTFL1, a constitutive local repressor of flowering, modulates floral initiation by antagonising florigen complex activity in chrysanthemum. *Plant Science* **237**, 1-7.

**Higuchi Y, Narumi T, Oda A, Nakano Y, Sumitomo K, Fukai S, Hisamatsu T**. 2013. The gated induction system of a systemic floral inhibitor, antiflorigen, determines obligate short-day flowering in chrysanthemums. *Proc Natl Acad Sci U S A* **110**, 17137-17142.

**Hodgins KA, Lai Z, Nurkowski K, Huang J, Rieseberg LH**. 2013. The molecular basis of invasiveness: differences in gene expression of native and introduced common ragweed (Ambrosia artemisiifolia) in stressful and benign environments. *Molecular Ecology* **22**, 2496-2510.

**Huang NC, Jane WN, Chen J, Yu TS**. 2012. Arabidopsis thaliana CENTRORADIALIS homologue (ATC) acts systemically to inhibit floral initiation in Arabidopsis. *The Plant Journal* **72**, 175-184.

**Igasaki T, Watanabe Y, Nishiguchi M, Kotoda N**. 2008. The FLOWERING LOCUS T/TERMINAL FLOWER 1 family in Lombardy poplar. *Plant and Cell Physiology* **49**, 291-300.

**Izawa T, Oikawa T, Sugiyama N, Tanisaka T, Yano M, Shimamoto K**. 2002. Phytochrome mediates the external light signal to repress FT orthologs in photoperiodic flowering of rice. *Genes & Development* **16**, 2006-2020.

**Kardailsky I, Shukla VK, Ahn JH, Dagenais N, Christensen SK, Nguyen JT, Chory J, Harrison MJ, Weigel D**. 1999. Activation tagging of the floral inducer FT. *science* **286**, 1962-1965.

**Kikuchi R, Kawahigashi H, Ando T, Tonooka T, Handa H**. 2009. Molecular and functional characterization of PEBP genes in barley reveal the diversification of their roles in flowering. *Plant Physiology* **149**, 1341-1353.

**Kojima S, Takahashi Y, Kobayashi Y, Monna L, Sasaki T, Araki T, Yano M**. 2002. Hd3a, a rice ortholog of the Arabidopsis FT gene, promotes transition to flowering downstream of Hd1 under short-day conditions. *Plant and Cell Physiology* **43**, 1096-1105.

**Komiya R, Ikegami A, Tamaki S, Yokoi S, Shimamoto K**. 2008. Hd3a and RFT1 are essential for flowering in rice. *Development* **135**, 767-774.

**Kong F, Liu B, Xia Z, Sato S, Kim BM, Watanabe S, Yamada T, Tabata S, Kanazawa A, Harada K, Abe J**. 2010. Two coordinately regulated homologs of FLOWERING LOCUS T are involved in the control of photoperiodic flowering in soybean. *Plant Physiology* **154**, 1220-1231.

**Koskela EA, Mouhu K, Albani MC, Kurokura T, Rantanen M, Sargent DJ, Battey NH, Coupland G, Elomaa P, Hytönen T**. 2012. Mutation in TERMINAL FLOWER1 reverses the photoperiodic requirement for flowering in the wild strawberry Fragaria vesca. *Plant Physiology* **159**, 1043-1054.

**Kotoda N, Hayashi H, Suzuki M, Igarashi M, Hatsuyama Y, Kidou S, Igasaki T, Nishiguchi M, Yano K, Shimizu T, Takahashi S, Iwanami H, Moriya S, Abe K**. 2010. Molecular characterization of FLOWERING LOCUS T-like genes of apple (Malus x domestica Borkh.). *Plant and Cell Physiology* **51**, 561-575.

**Kotoda N, Iwanami H, Takahashi S, Abe K**. 2006. Antisense expression of MdTFL1, a TFL1-like gene, reduces the juvenile phase in apple. *Journal of the American Society for Horticultural Science* **131**, 74-81.

**Laurie RE, Diwadkar P, Jaudal M, Zhang L, Hecht V, Wen J, Tadege M, Mysore KS, Putterill J, Weller JL, Macknight RC**. 2011. The Medicago FLOWERING LOCUS T homolog, MtFTa1, is a key regulator of flowering time. *Plant Physiology* **156**, 2207-2224.

**Lee R, Baldwin S, Kenel F, McCallum J, Macknight R**. 2013. FLOWERING LOCUS T genes control onion bulb formation and flowering. *Nat Commun* **4**, 2884.

**Li X-M, Zhang D-Y, Liao W-J**. 2015. The rhythmic expression of genes controlling flowering time in southern and northern populations of invasive Ambrosia artemisiifolia. *Journal of Plant Ecology* **8**, 207-212.

**Lifschitz E, Eviatar T, Rozman A, Shalit A, Goldshmidt A, Amsellem Z, Alvarez JP, Eshed Y**. 2006. The tomato FT ortholog triggers systemic signals that regulate growth and flowering and substitute for diverse environmental stimuli. *Proc Natl Acad Sci U S A* **103**, 6398-6403.

**Lin MK, Belanger H, Lee YJ, Varkonyi-Gasic E, Taoka K, Miura E, Xoconostle-Cazares B, Gendler K, Jorgensen RA, Phinney B, Lough TJ, Lucas WJ**. 2007. FLOWERING LOCUS T protein may act as the long-distance florigenic signal in the cucurbits. *Plant Cell* **19**, 1488-1506.

**Liu B, Watanabe S, Uchiyama T, Kong F, Kanazawa A, Xia Z, Nagamatsu A, Arai M, Yamada T, Kitamura K, Masuta C, Harada K, Abe J**. 2010. The soybean stem growth habit gene Dt1 is an ortholog of Arabidopsis TERMINAL FLOWER1. *Plant Physiology* **153**, 198-210.

**Meng X, Muszynski MG, Danilevskaya ON**. 2011. The FT-like ZCN8 Gene Functions as a Floral Activator and Is Involved in Photoperiod Sensitivity in Maize. *Plant Cell* **23**, 942-960.

**Mimida N, Kotoda N, Ueda T, Igarashi M, Hatsuyama Y, Iwanami H, Moriya S, Abe K**. 2009. Four TFL1/CEN-like genes on distinct linkage groups show different expression patterns to regulate vegetative and reproductive development in apple (Malus x domestica Borkh.). *Plant and Cell Physiology* **50**, 394-412.

**Mohamed R, Wang CT, Ma C, Shevchenko O, Dye SJ, Puzey JR, Etherington E, Sheng X, Meilan R, Strauss SH, Brunner AM**. 2010. Populus CEN/TFL1 regulates first onset of flowering, axillary meristem identity and dormancy release in Populus. *The Plant Journal* **62**, 674-688.

**Nakagawa M, Shimamoto K, Kyozuka J**. 2002. Overexpression of RCN1 and RCN2, rice TERMINAL FLOWER 1/CENTRORADIALIS homologs, confers delay of phase transition and altered panicle morphology in rice. *The Plant Journal* **29**, 743-750.

**Navarro C, Abelenda JA, Cruz-Oro E, Cuellar CA, Tamaki S, Silva J, Shimamoto K, Prat S**. 2011. Control of flowering and storage organ formation in potato by FLOWERING LOCUS T. *Nature* **478**, 119-122.

**Oda A, Narumi T, Li T, Kando T, Higuchi Y, Sumitomo K, Fukai S, Hisamatsu T**. 2012. CsFTL3, a chrysanthemum FLOWERING LOCUS T-like gene, is a key regulator of photoperiodic flowering in chrysanthemums. *Journal of Experimental Botany* **63**, 1461-1477.

**Pin PA, Benlloch R, Bonnet D, Wremerth-Weich E, Kraft T, Gielen JJ, Nilsson O**. 2010. An antagonistic pair of FT homologs mediates the control of flowering time in sugar beet. *science* **330**, 1397-1400.

**Pnueli L, Carmel-Goren L, Hareven D, Gutfinger T, Alvarez J, Ganal M, Zamir D, Lifschitz E**. 1998. The SELF-PRUNING gene of tomato regulates vegetative to reproductive switching of sympodial meristems and is the ortholog of CEN and TFL1. *Development* **125**, 1979-1989.

**Repinski SL, Kwak M, Gepts P**. 2012. The common bean growth habit gene PvTFL1y is a functional homolog of Arabidopsis TFL1. *Theoretical and Applied Genetics* **124**, 1539-1547.

**Varkonyi-Gasic E, Moss S, Voogd C, Wang T, Putterill J, Hellens RP**. 2013. Homologs of FT, CEN and FD respond to developmental and environmental signals affecting growth and flowering in the perennial vine kiwifruit. *New Phytologist* **198**, 732-746.

**Voogd C, Brian LA, Wang T, Allan AC, Varkonyi-Gasic E**. 2017. Three FT and multiple CEN and BFT genes regulate maturity, flowering, and vegetative phenology in kiwifruit. *Journal of Experimental Botany* **68**, 1539-1553.

**Xiang L, Li X, Qin D, Guo F, Wu C, Miao L, Sun C**. 2012. Functional analysis of FLOWERING LOCUS T orthologs from spring orchid (Cymbidium goeringii Rchb. f.) that regulates the vegetative to reproductive transition. *Plant Physiology and Biochemistry* **58**, 98-105.

**Yamaguchi A, Kobayashi Y, Goto K, Abe M, Araki T**. 2005. TWIN SISTER OF FT (TSF) acts as a floral pathway integrator redundantly with FT. *Plant and Cell Physiology* **46**, 1175-1189.

**Yan L, Fu D, Li C, Blechl A, Tranquilli G, Bonafede M, Sanchez A, Valarik M, Yasuda S, Dubcovsky J**. 2006. The wheat and barley vernalization gene VRN3 is an orthologue of FT. *Proc Natl Acad Sci U S A* **103**, 19581-19586.

**Ye J, Geng Y, Zhang B, Mao H, Qu J, Chua N-H**. 2014. The Jatropha FT ortholog is a systemic signal regulating growth and flowering time. *Biotechnology for Biofuels* **7**, 91.

**Yoo SJ, Chung KS, Jung SH, Yoo SY, Lee JS, Ahn JH**. 2010. BROTHER OF FT AND TFL1 (BFT) has TFL1-like activity and functions redundantly with TFL1 in inflorescence meristem development in Arabidopsis. *The Plant Journal* **63**, 241-253.

**Zhang S, Hu W, Wang L, Lin C, Cong B, Sun C, Luo D**. 2005. TFL1/CEN-like genes control intercalary meristem activity and phase transition in rice. *Plant Science* **168**, 1393-1408.
